# Uncovering an antifibrotic *Prrx1*-lineage mesenchymal cell subpopulation in fibrotic lungs

**DOI:** 10.1242/dmm.052179

**Published:** 2025-08-26

**Authors:** Meline Homps-Legrand, Madeleine Jaillet, Lou Deneuville, Gregory Gautier, Bruno Crestani, Arnaud A. Mailleux

**Affiliations:** ^1^ Université Paris Cité, Inserm, Centre de Recherche sur l'Inflammation, F-75018 Paris, France; ^2^ Hôpital Bichat, AP-HP, Service de Pneumologie, Allergologie et Transplantation, Centre Constitutif du Centre de Référence des Maladies Pulmonaires Rares, FHU INFIRE, 75018 Paris, France

**Keywords:** Lung fibrosis, Transcription factors, Fibroblast, Bleomycin, Lineage tracing

## Abstract

Idiopathic pulmonary fibrosis (IPF) is a rare and fatal lung disease caused by progressive damage to alveolar epithelial cells, leading to abnormal activation of mesenchymal cells. The PRRX1 transcription factor (TF) has been found to be reactivated in IPF and was previously identified as a key mesenchymal TF in pulmonary fibrosis. In this study, we utilized the *Prrx1:Cre^ERT2^; Rosa26iTomato* murine transgenic line to further characterize the *Prrx1*-positive cell lineage in healthy and fibrotic lungs. The *Prrx1* limb enhancer (*Prrx1^enh^*) was undetectable by immunohistochemistry in uninjured lung tissue. However, during the fibrotic phase in the bleomycin model of pulmonary fibrosis, *Prrx1^enh^* became activated, marking a population of cells that differentiated into mesenchymal progeny. To investigate further, we conducted reprogramming of these subpopulations after conditional and inducible *Prrx1* loss of function. *Prrx1* loss in these cells led to worsened fibrosis, indicating that this specific cell population has antifibrotic properties. Our findings reveal a previously unrecognized subpopulation of *Prrx1-*positive mesenchymal cells that are activated during fibrogenesis. These cells could serve as targets for future therapies aimed at mitigating fibrotic progression in IPF.

## Teaser

**Summary:** This study reveals a novel mesenchymal cell subpopulation, identified by the *Prrx1* transcription factor, that emerges during lung fibrosis in mice and exhibits protective, anti-scarring properties.

## INTRODUCTION

Idiopathic pulmonary fibrosis (IPF) is a rare, chronic and progressive lung disease associated with poor prognosis (Lederer and Martinez, 2018). IPF falls under the category of interstitial lung diseases, characterized by significant alterations in the lung parenchyma leading to major gas exchange defects (Martinez et al., 2017). Currently, the two approved medications, Nintedanib and Pirfenidone, only serve to slow down the progression of IPF (Kistler et al., 2014).

The current pathophysiological hypothesis suggests that IPF is primarily driven by the epithelium, with aberrant remodelling attempting to compensate for chronic alveolar epithelial injuries (Álvarez et al., 2017; Homps-Legrand et al., 2023; Plantier et al., 2018). These processes are associated with the reactivation of lung developmental pathways (Chanda et al., 2019; Froidure et al., 2020), including TGF-β, Wnt and FGF pathways, as well as transcription factors (TFs) such as PRRX1 (Marchal-Duval et al., 2023).

PRRX1 was initially identified for its critical role in lung and limb development. *Prrx1*^−/−^ pups exhibit craniofacial abnormalities (Martin et al., 1995), skeletal dysgenesis (ten Berge et al., 1998) and lung vasculature abnormalities (Ihida-Stansbury et al., 2004), resulting in neonatal lethality. PRRX1 expression is disrupted in various pathologies, including craniofacial diseases (Sergi and Kamnasaran, 2011), cancers (Ocaña et al., 2012), and liver and skin fibrosis (Currie et al., 2019).

Recently, lineage tracing using a *Prrx1:CreERT2* mouse line demonstrated that a *Prrx1*-positive mesenchymal cell population – referred to as the *Prrx1^enh^* subpopulation – was amplified and associated with fibrosis development after skin injury in the mouse ventral dermis (Currie et al., 2019; Leavitt et al., 2020), and it modulated skin inflammation during atopic dermatitis (Ko et al., 2022). This *Prrx1* mesenchymal subpopulation was recently found to play a role in scarless repair within the oral mucosa (Ko et al., 2023). Recent studies indicated that PRRX1 TF controls the phenotype of a subset of cancer-associated fibroblasts promoting tumour growth and survival (Feldmann et al., 2021; Lee et al., 2022).

With regard to lung fibrosis, our group identified PRRX1 as a key mesenchymal TF during fibrosis development (Marchal-Duval et al., 2023). PRRX1 is expressed in fibroblasts within perivascular and peribronchiolar spaces of healthy human lungs. We, and others, reported nuclear expression of PRRX1 in the mesenchymal cells forming fibroblastic foci in patients with IPF (Marchal-Duval et al., 2023; Yeo et al., 2018). *In vitro* studies demonstrated that PRRX1 exhibited profibrotic features by promoting fibroblast proliferation and TGF-β-driven myofibroblast differentiation. *In vivo* experiments showed that global *Prrx1* inhibition using a *Prrx1*-targeting antisense oligonucleotide (ASO) significantly attenuated fibrosis in the murine bleomycin model of pulmonary fibrosis (Marchal-Duval et al., 2023).

In the present study, we further characterized the *Prrx1*-positive mesenchymal cells in the bleomycin preclinical mouse model of lung fibrosis using a lineage tracing approach (Homps-Legrand et al., 2023) with a *Prrx1:Cre^ERT2^; Rosa26iTomato* transgenic mouse line (Kawanami et al., 2009). Additionally, we investigated the functional impact of this population in healthy and fibrotic lungs after conditional and inducible loss of *Prrx1* using a newly generated floxed allele (*Prrx1^fl^*).

## RESULTS

### *Prrx1^enh^*-positive cells are sparse in the lung at steady state

**Fig. 1.**
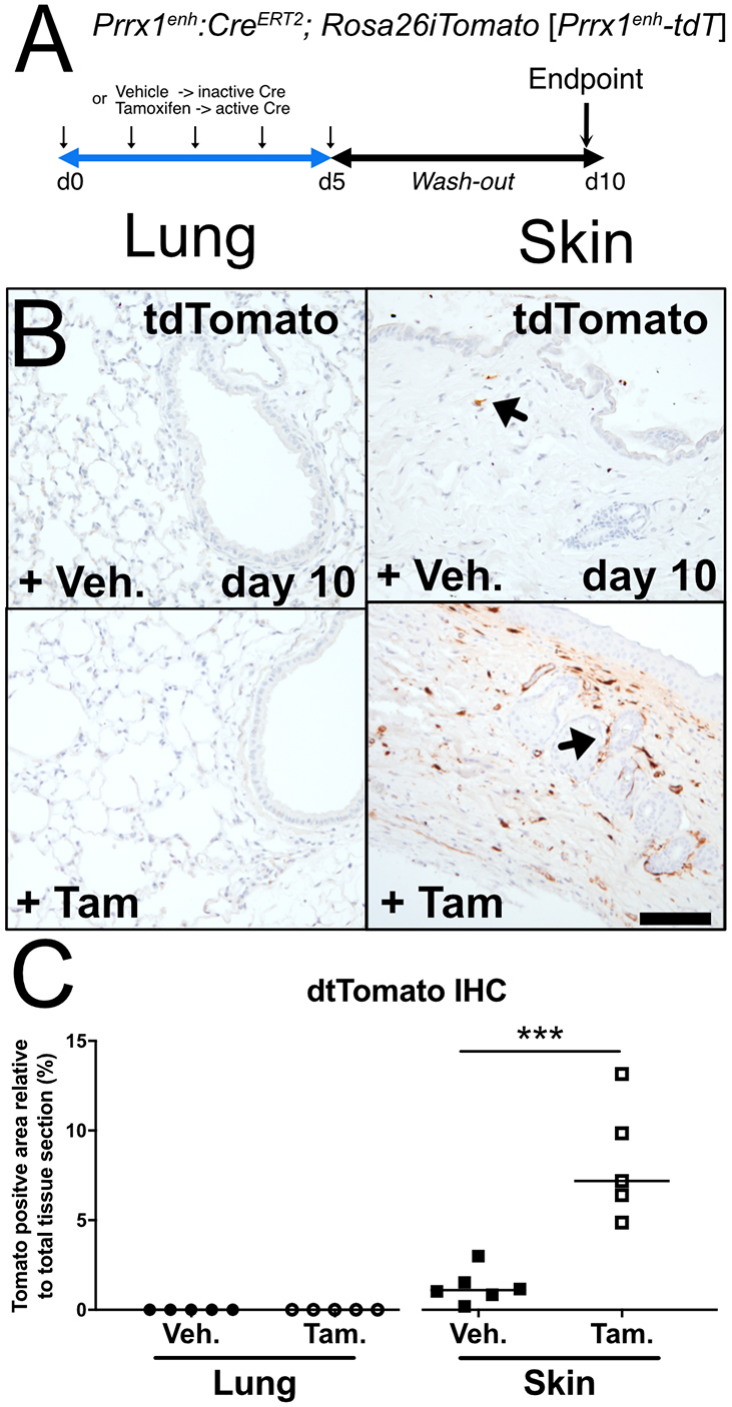
**Identification of cells expressing the *Prrx1^enh^* promoter in the lung and ventral skin at baseline.** (A) Timeline of vehicle and tamoxifen treatments. *Prrx1:Cre^ERT2^; Rosa26iTomato* naive (*Prrx1^enh^-tdT*) mice were injected with vehicle or tamoxifen every day for 5 days. Lungs and ventral skin were collected at Day 10 after a wash-out time of 5 days. (B) Representative tdTomato immunohistochemistry (*n*=5 to 6 per group, brown staining) in *Prrx1^enh^-tdT* mouse lungs (left column) or ventral skin (right column) treated with either vehicle (top row) or tamoxifen (bottom row). Nuclei were counterstained with Haematoxylin. Left column: note the absence of tdTomato staining in the lungs collected from both vehicle- and tamoxifen-treated animals. Right column: note the few tdTomato-positive areas in the vehicle-treated animals (Cre leakage) compared to the numerous tdTomato-positive areas in the tamoxifen-treated group. Scale bar: 50 µm. (C) Quantification of the area of tdTomato-positive cells (% of section) in the lung (circles; *n*=5) and skin (squares; *n*=5 to 6) after vehicle (filled) or tamoxifen (open) treatment; data shown as dot plots with median. Nuclei were counterstained with Haematoxylin. Mann–Whitney U-test: ***P≤0.001. The absence of statistical labelling indicates non-significative results.

At baseline, *Prrx1* expression was previously reported as restricted to the fibroblast lineage in perivascular and peribronchiolar spaces in the lung (Marchal-Duval et al., 2023). First, lineage tracing was performed to label pre-existing *Prrx1*-positive cells in the lung using *Prrx1:Cre^ERT2^; Rosa26iTomato* (*Prrx1^enh^-tdT*) naive mice. In this previously generated Cre mouse line (Kawanami et al., 2009), the Cre^ERT2^ recombinase is under the control of a 2.4 kb *Prrx1* partial promoter cassette, also known as *Prrx1* limb enhancer (*Prrx1^enh^*). Animals were injected with tamoxifen or vehicle only (corn oil) for 5 days, and tissue samples were collected after 5 days of wash-out to ensure enough time for tamoxifen clearance (Fig. 1A). Immunohistochemistry (IHC) using an anti-RFP antibody (which also recognizes tdTomato) was performed on formalin-fixed paraffin-embedded (FFPE) lung sections to label *Prrx1^enh^*-positive cell populations (Fig. 1B,C). Surprisingly, no tdTomato-positive cells were detected in the lung (Fig. 1B,C), whereas numerous such cells were observed in the ventral skin (Fig. 1B), albeit with some degree of leakage (Fig. 1B,C), as previously reported (Currie et al., 2019). In the lung, the absence of detectable labelling with the *Prrx1^enh^-Cre* driver contrasts with the presence of PRRX1-positive cells, including PI16-positive adventitial fibroblasts, identified by IHC in both vehicle- and tamoxifen-treated tissue (Fig. S1). Together, these results suggest that either the *Prrx1^enh^* element is not active in the lung or the *Prrx1^enh^*-positive cells constitute a very rare subpopulation of *Prrx1*-expressing cells that is difficult to detect by IHC on FFPE lung sections of healthy adult mice.

### The *Prrx1^enh^*-positive cell population is amplified during the fibrotic phase of pulmonary fibrosis after bleomycin challenge

**Fig. 2.**
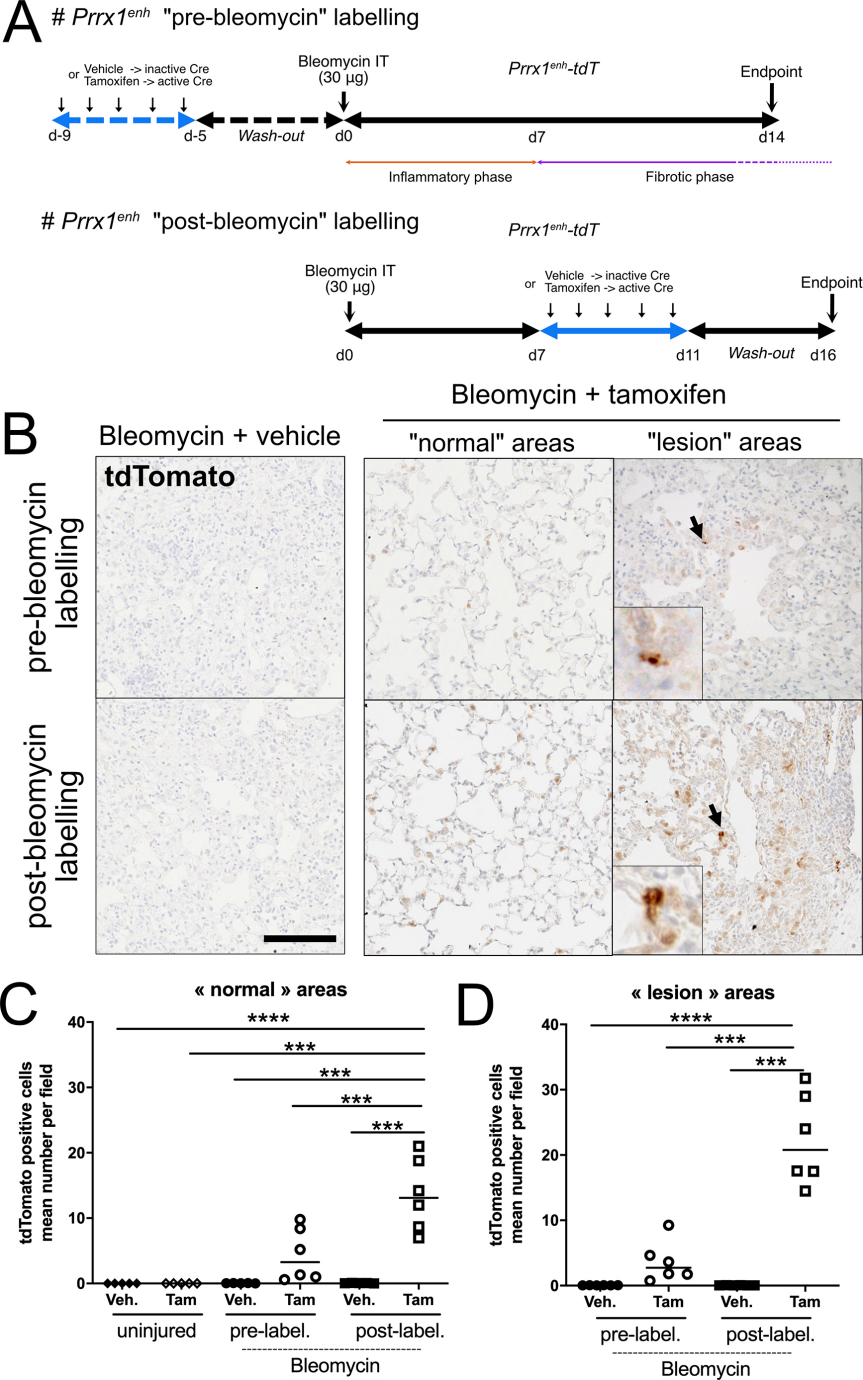
**Identification of a *Prrx1^enh^*-positive subpopulation activated during lung fibrosis.** (A) Timeline of vehicle and tamoxifen treatments. Top: ‘pre-bleomycin’ labelling protocol in *Prrx1^enh^-tdT* mice: intraperitoneal injections of corn oil only (vehicle) or tamoxifen were performed from Day −10 to Day −5 before receiving a single intratracheal dose of bleomycin (30 μg) at Day 0. Lung samples were collected at Day 14 after bleomycin treatment. Bottom: ‘post-bleomycin’ labelling protocol in *Prrx1^enh^-tdT* mice: intraperitoneal injections of corn oil only (vehicle) or tamoxifen were performed from Day 7 to Day 11 after receiving a single intratracheal dose of bleomycin (30 μg) at Day 0. Lung samples were collected at Day 16 after a 5-day wash-out. d, day; IT, intratracheal. (B) Representative tdTomato immunohistochemistry (*n*=5-6 per group, brown staining) in *Prrx1^enh^-tdT* mouse fibrotic lungs following pre-bleomycin (Day 14) or post-bleomycin (Day 16) protocols after vehicle or tamoxifen treatments. Note the absence of tdTomato-positive cells in vehicle-treated animals. Arrows indicate the areas shown at high magnification in the insets. Nuclei were counterstained with Haematoxylin. Scale bar: 50 µm. (C) Quantification of tdTomato-positive cells in ‘normal’ lung areas from mice after pre-bleomycin (circles; *n*=5) and post-bleomycin (squares; *n*=5) labelling protocols as well as in uninjured lungs (diamonds; *n*=5; see protocol in Fig. 1A) after vehicle (filled; *n*=6) or tamoxifen (open; *n*=6) treatments; data shown as dot plots with median. (D) Quantification of tdTomato-positive cells in ‘lesion’ lung areas from mice following pre-bleomycin (circles; *n*=5) and post-bleomycin (squares; *n*=5) labelling protocols after vehicle (filled; *n*=6) or tamoxifen (open; *n*=6) treatments; data shown as dot plots with median. One-way ANOVA with Tukey's comparison test: ***P≤0.01, ****P≤0.0005. The absence of statistical labelling indicates non-significative results.

To study the activity of the *Prrx1^enh^* element during lung fibrosis, we undertook two different experimental protocols (Fig. 2A). In the first protocol (‘pre-bleomycin’ labelling), mice were first treated with tamoxifen or vehicle for 5 days, followed by a 5-day wash-out period, before a unique intratracheal instillation of bleomycin (Fig. 2A). This approach was designed to label any *Prrx1*^enh^-positive cells present in the lung prior to injury and assess their potential involvement in the fibrotic response at Day 14. However, given the scarcity or possible absence of detectable *Prrx1^enh^* activity in the healthy lung, this strategy primarily served to test whether these cells expand or become more apparent following fibrotic injury. In a second protocol (‘post-bleomycin’ labelling), a unique dose of bleomycin was first instilled. Animals were next treated with tamoxifen (bleomycin+tamoxifen group) or vehicle (bleomycin+vehicle group) from Day 7 to Day 11 to label *Prrx1^enh^*-positive cells during the fibrotic phase of this mouse model of pulmonary fibrosis (Fig. 2A). The fate of those cells was analysed at Day 16 to allow a 5-day wash-out period.

We next performed tdTomato IHC on paraffin lung sections to label the progeny of the *Prrx1^enh^*-positive cells in both protocols (Fig. 2B). We observed tdTomato-positive cells in both protocols, with a higher number in the post-bleomycin protocol compared to that in the pre-bleomycin one (Fig. 2B,C). We next performed tdTomato IHC on paraffin-embedded lung sections to detect the progeny of *Prrx1*^enh^-positive cells in both protocols (Fig. 2B). Cells expressing tdTomato were observed in both experimental groups, with a higher number detected in the post-bleomycin protocol compared to that in the pre-bleomycin protocol (Fig. 2B-D). For instance, we detected a 4.4-fold increase in the number of tdTomato-positive cells in ‘normal’-looking areas and a 10-fold increase in the fibrotic territories (as defined in the Material and Methods section) of bleomycin+tamoxifen-treated animals in the post-bleomycin protocol (tamoxifen pulse during the fibrotic phase) compared to the pre-bleomycin regimen (Fig. 2C,D).

Overall, these results suggest that the activity of the *Prrx1* limb enhancer (*Prrx1^enh^*) is only detectable by IHC after an injury in the lung. Based on these lineage tracing results, we decided to further characterize the progeny of the *Prrx1^enh^*-positive cells in the post-bleomycin protocol, associated with true amplification of this population during lung fibrogenesis.

### *Prrx1^enh^*-positive cell progeny display mesenchymal features during lung fibrosis

**Fig. 3.**
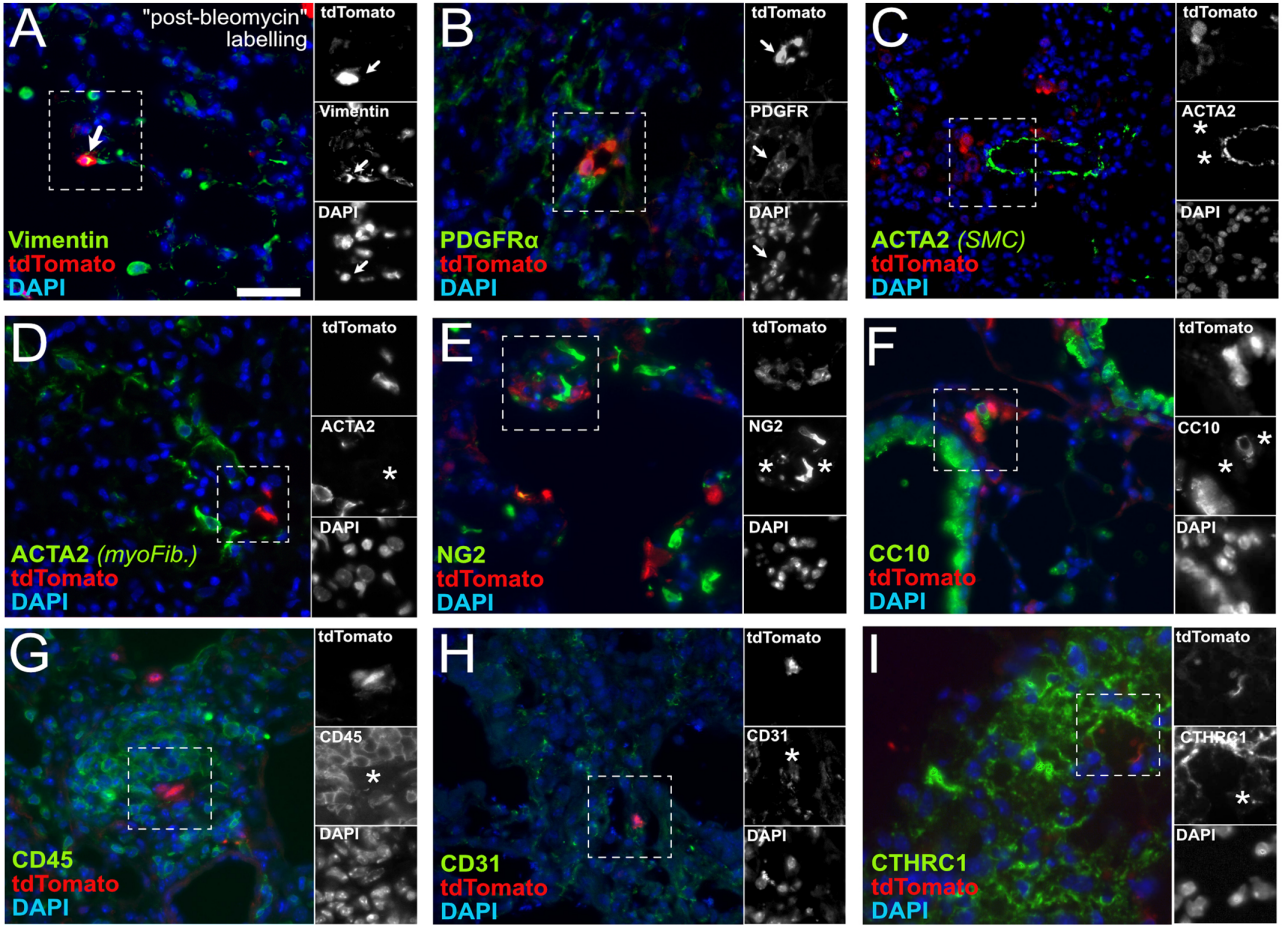
**Co-immunostaining of tdTomato with lung cell markers in *Prrx1^enh^-tdT* lungs after post-bleomycin labelling.** (A-I) Multiplex immunofluorescence of tdTomato (red signal) and the relative percentage of tdTomato-positive cells for the following markers (green signal) – vimentin (100% positive) (A), PDGFRα (100% positive) (B), ACTA2 in smooth muscle cells (0% positive) (C), ACTA2 in myofibroblasts (0% positive) (D), NG2 (0% positive) (E), CC10 (0% positive) (F), CD45(0% positive) (G), CD31 (0% positive) (H) and CTHRC1 (0% positive) (I) – in *Prrx1^enh^-tdT* mouse fibrotic lungs (*n*=5 animals) treated with tamoxifen following the post-bleomycin protocol. Separate channels in greyscale are presented in the insets (areas in dashed line boxes). DAPI (blue) was used for nuclei staining. Arrows and asteriks indicate the position of the tdTomato-positive cells in the panel and the corresponding insets. Scale bar: 50 µm. myoFib., myofibroblasts; SMC, smooth muscle cells.

In order to characterize the tdTomato-positive cells accumulating after a fibrotic insult, we performed double immunofluorescence staining with tdTomato and several general lung cell lineage markers (Kheirollahi et al., 2019; Rock et al., 2011): vimentin (mesenchymal lineage), PDGFRα (fibroblast subpopulation), ACTA2 (smooth muscle cells and myofibroblasts), NG2 (also known as CSPG4; pericytes), CC10 (also known as SCGB1A1; club cells), CD45 (also known as PTPRC; hematopoietic lineage), CD31 (also known as PECAM1; endothelial cells) and CTHRC1 (myofibroblast subpopulation). We observed colocalization of tdTomato with vimentin and PDGFRα staining (Fig. 3A,B), whereas no colocalization was observed with ACTA2 (neither in smooth muscle cells nor in myofibroblasts in remodelled areas; Fig. 3C,D), NG2, CC10, CD45, CD31 and CTHRC1 (Fig. 3D-I). These results suggest that the *Prrx1^enh^*-positive cells labelled during the fibrotic phase after bleomycin challenge gave rise to CD45^neg^ CD31^neg^ ACTA2^neg^ CTHRC1^neg^ VIM^pos^ PDGFRα^pos^ (neg, negative; pos, positive) mesenchymal cells, representing a composite sketch of fibroblasts.

### Inducible *Prrx1* loss of function in *Prrx1^enh^*-positive cells is not overall detrimental in the lung at baseline

Given that *Prrx1* constitutive loss of function results in perinatal lethality in *Prrx1^−/−^* mice (Martin et al., 1995), we generated a *Prrx1* floxed allele (*Prrx1^fl^*) to investigate the role of *Prrx1* in adult animals. This transgenic mouse line was designed with exon 2 of the *Prrx1* gene flanked by two *LoxP* sequences (Fig. S2). To validate the newly generated *Prrx1* floxed allele, we derived immortalized mouse embryonic fibroblasts (MEFs) from *Prrx1^fl^; Rosa26iTomato* embryos and infected them with Cre adenovirus *in vitro* to allow the excision of the exon 2 of *Prrx1* gene. The expression of tdTomato served as a marker of Cre-mediated recombination events in the transduced cells. Upon subcloning of tdTomato-positive MEFs, we confirmed the successful excision of *Prrx1* exon 2 by PCR analysis (Fig. S2). Western blot analysis and immunofluorescence staining of PRRX1 and tdTomato (Fig. S2) further validated *Prrx1* loss of function in these cells compared to the parental ones.

*Prrx1:Cre^ERT2^; Prrx1^fl/fl^; Rosa26iTomato* (*Prrx1^enh^-tdT-cKO*) animals were generated to enable targeted and inducible *Prrx1* loss of function specifically in the *Prrx1^enh^* subpopulation and simultaneously label this subpopulation with tdTomato. As shown in Fig. 1, lineage tracing experiments indicated a lack of labelling efficiency of the *Prrx1^enh^* transgene in uninjured lungs. To confirm the lack of influence of the *Prrx1^enh^* element in the uninjured lung and to evaluate the impact of *Prrx1* loss specifically in this model, *Prrx1^enh^*-*tdT-cKO* mice were treated with tamoxifen or vehicle alone for 5 days, then analysed after a 5-day wash-out period. We did not observe any discernible effects of *Prrx1* loss in *Prrx1^enh^*-expressing cells on lung histology or bronchoalveolar lavage (BAL) cellularity at baseline (Fig. S3). Further investigation of mRNA expression of fibrosis-associated genes (*Col1a1*, *Fn1* and *Acta2*) and the overall levels of the two *Prrx1* isoforms (*Prrx1a* and *Prrx1b*) revealed no significant differences between the two groups (Fig. S3). At the protein level, there were no significant changes in the expression levels of PRRX1, extracellular matrix (ECM) components (COL1 and FN1), and the smooth muscle and myofibroblast marker ACTA2 between vehicle and tamoxifen-treated animals (Fig. S3). Moreover, no significant difference was detected in the mRNA expression of other profibrotic genes [*Col3a1*, *Col14a1*, *Eln*, *Tgfb1*, *Pai1* (also known as *Serpine1*) and *Ctgf* (also known as *Ccn2*)] (Fig. S4). However, we observed a noteworthy increase in *Tnc* mRNA levels in the tamoxifen-treated group compared to those in the vehicle group. Interestingly, we found significant upregulation of the antifibrotic gene *Fgf7* at the mRNA level in the tamoxifen-treated group, whereas no difference was observed for the other antifibrotic genes (*Hgf* and *Fgf10*) (Fig. S3). Importantly, this increase in *Fgf7* and *Tnc* mRNA expression levels was not a consequence of tamoxifen treatment, as it was not observed in the lungs of *Prrx1^enh^-tdT* mice treated with tamoxifen compared to those treated with vehicle (M.H.-L., data not shown). Taken together, these results indicate that although *Prrx1* loss using a *Prrx1^enh^-Cre* driver population did not visibly affect overall lung architecture and fibrosis markers expression, it was sufficient to influence basal *Fgf7* mRNA expression even in a non-fibrotic setting.

### Inducible *Prrx1* loss of function in *Prrx1^enh^*-positive cells during the fibrotic phase worsens lung fibrosis after bleomycin insult

**Fig. 4.**
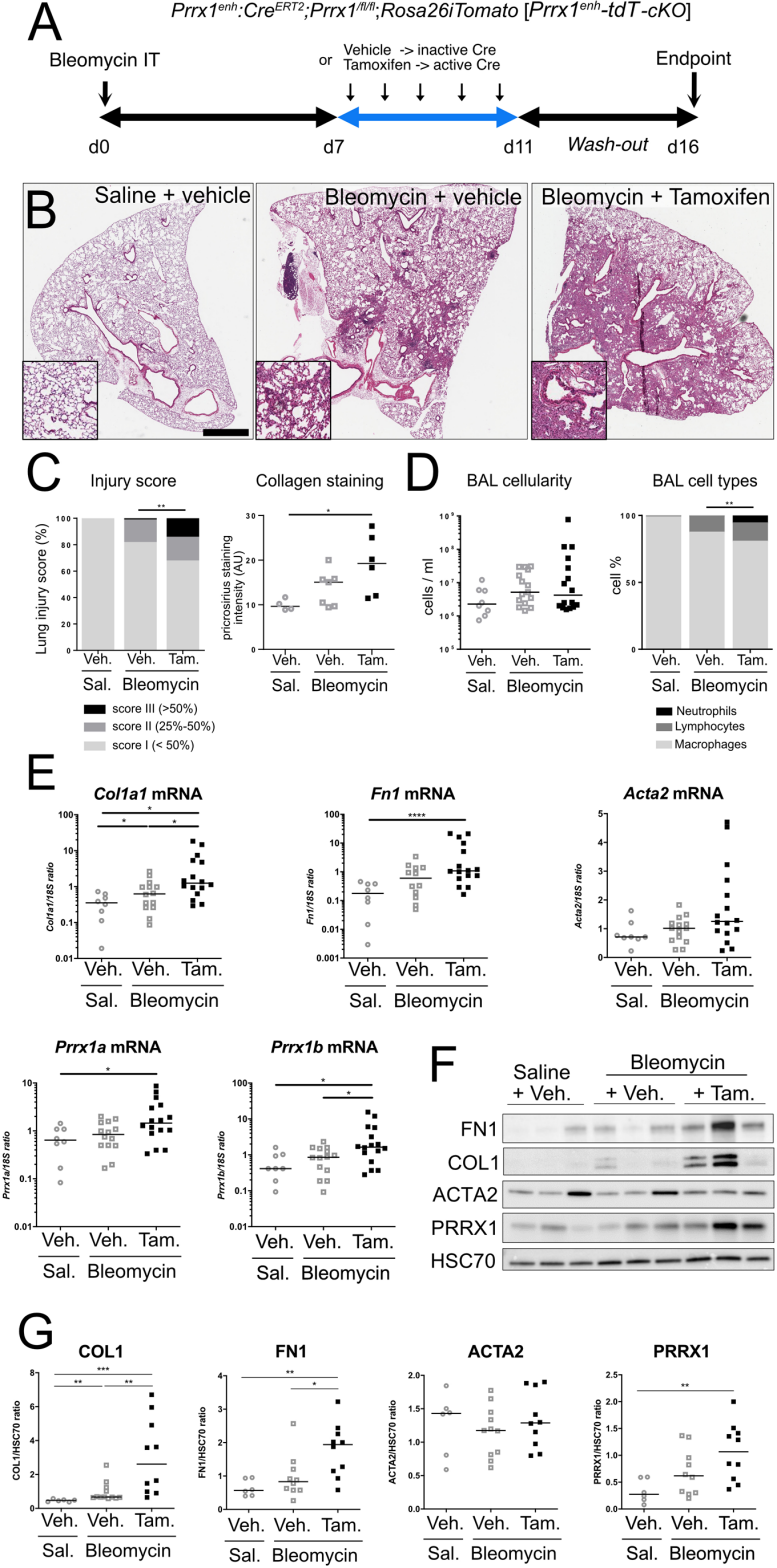
***Prrx1* loss of function in *Prrx1^enh+^* cells worsens bleomycin-induced lung fibrosis.** (A) Timeline of vehicle and tamoxifen treatments. *Prrx1:Cre^ERT2^; Rosa26iTomato; Prrx1^fl/fl^* mice (*Prrx1^enh^-tdT-cKO*) mice underwent intraperitoneal injections of corn oil only (vehicle) or tamoxifen from Day 7 to Day 11 after receiving a single intratracheal dose of bleomycin (30 μg) at Day 0. Lung samples were collected at Day 16 after a 5-day wash-out. (B) Representative images of Haematoxylin and Eosin staining of lung sections from saline+vehicle-, bleomycin+vehicle- or bleomycin+tamoxifen-treated *Prrx1^enh^-tdT-cKO* mice (at least *n*=5 per group). High-magnification pictures are displayed in the insets. Scale bar: 50 µm. (C) Left: injury score at Day 16 of saline- and bleomycin-treated mice also treated with vehicle or tamoxifen (at least *n*=7 per group). Right: quantification of Picrosirius Red intensity (associated with collagen deposition; at least *n*=7 per group) in saline+vehicle (open circles), bleomycin+vehicle (open squares) and bleomycin+tamoxifen (filled squares) groups. Note that all bleomycin-treated animals in both groups displayed lesions compared to saline-treated animals. (D) Left: bronchoalveolar lavage (BAL) cell count in saline+vehicle (open circles), bleomycin+vehicle (open squares) and bleomycin+tamoxifen (filled squares) groups (at least *n*=7 per group). Right: quantification of BAL cell types – neutrophils (black), lymphocytes (dark grey) and macrophages (light grey) – in saline+vehicle, bleomycin+vehicle and bleomycin+tamoxifen groups (at least *n*=7 per group). (E) Dot plots with median showing the mRNA expression of *Col1a1*, *Fn1*, *Acta2*, *Prrx1a* and *Prrx1b* in whole-lung extracts of mice after receiving saline+vehicle (open circles; *n*=8), bleomycin+vehicle (open squares; *n*=15) or bleomycin+tamoxifen (filled squares; *n*=17). (F) Representative immunoblot showing PRRX1, COL1, FN1 and ACTA2 expression in whole-lung extracts of mice after receiving saline+vehicle, bleomycin+vehicle or bleomycin+tamoxifen following the post-bleomycin protocol. HSC70 was used as loading control. (G) Quantification of PRRX1, COL1, FN1 and ACTA2 expression (relative to HSC70 in F) in saline+vehicle (open circles; *n*=6), bleomycin+vehicle (open squares; *n*=10) and bleomycin+tamoxifen (filled squares; *n*=10) groups; data shown as dot plots with median. Chi-square test (left panels in C and D), one-way ANOVA with Tukey's comparison test (right panels in C and D; E and G): *P≤0.05, **P≤0.01, ***P≤0.001, ****P≤0.0005. The absence of statistical labelling indicates non-significative results. AU, arbitrary units.

**Fig. 5.**
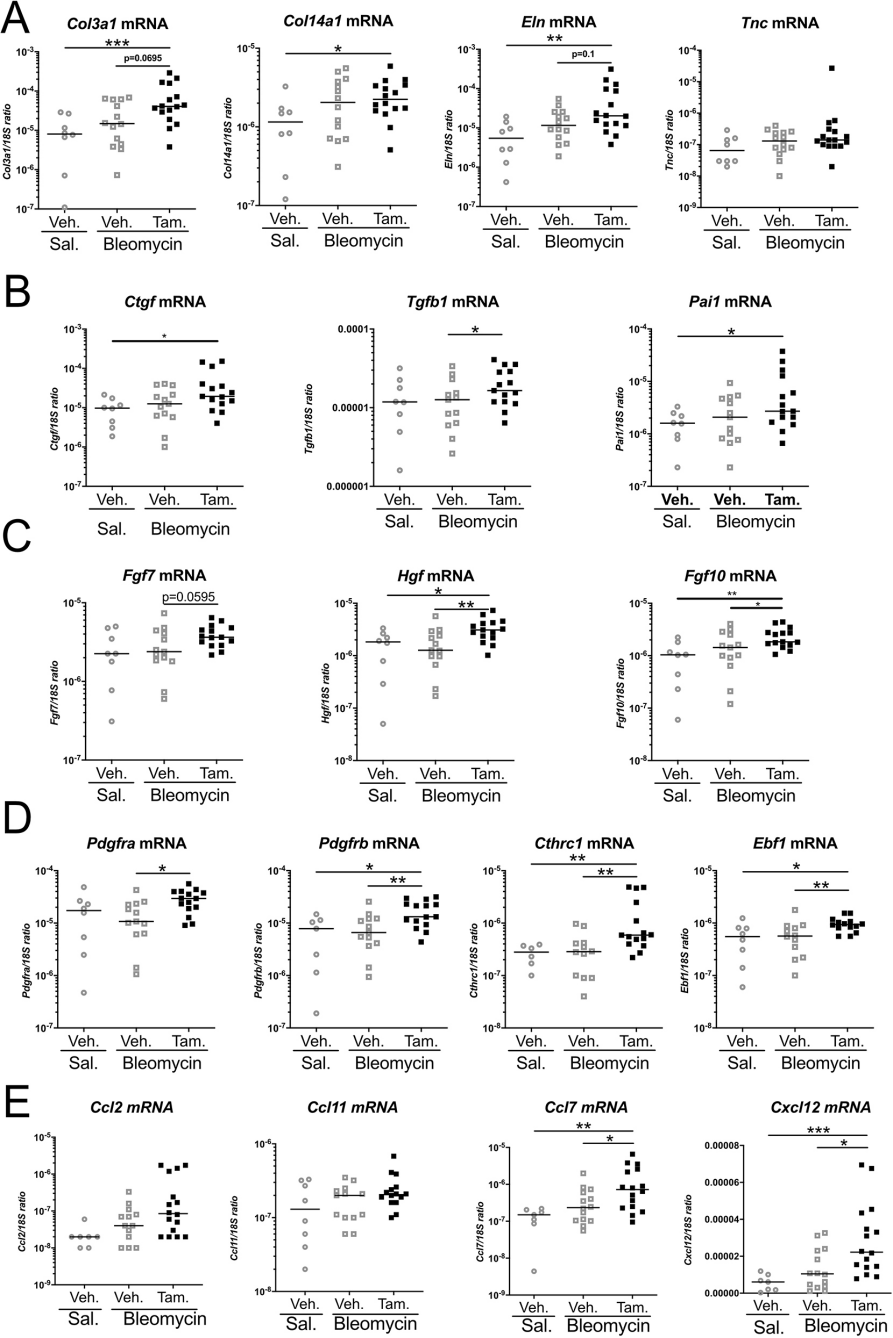
**mRNA expression of lung fibrosis-associated markers after *Prrx1* loss in *Prrx1^enh^*-positive cells after bleomycin-induced lung fibrosis.** (A) Dot plots with median showing the mRNA expression of the profibrotic markers *Col3a1*, *Col14a1*, *Tnc* and *Eln* in whole-lung extracts from *Prrx1^enh^-tdT-cKO* animals after receiving saline+vehicle (open circles; *n*=8), bleomycin+vehicle (open squares; *n*=15) and bleomycin+tamoxifen (filled squares; *n*=17). (B) Dot plots with median showing the mRNA expression of the profibrotic secreted markers *Ctgf*, *Tgfb1* and *Pai1* in whole-lung extracts of *Prrx1^enh^-tdT-cKO* mice after receiving saline+vehicle (open circles), bleomycin+vehicle (open squares) and bleomycin+tamoxifen (filled squares). (C) Dot plots with median showing the mRNA expression of the profibrotic markers *Fgf7*, *Hgf* and *Fgf10* in whole-lung extracts of *Prrx1^enh^-tdT-cKO* mice after receiving saline+vehicle (open circles), bleomycin+vehicle (open squares) and bleomycin+tamoxifen (filled squares). (D) Dot plots with median showing the mRNA expression of the profibrotic markers *Pdgfra*, *Pdgfrb*, *Cthrc1* and *Ebf1* in whole-lung extracts of *Prrx1^enh^-tdT-cKO* mice after receiving saline+vehicle (open circles), bleomycin+vehicle (open squares) and bleomycin+tamoxifen (filled squares). (E) Dot plots with median showing the mRNA expression of *Ccl2*, *Ccl11*, *Ccl7* and *Cxcl12* cytokines in whole-lung extracts of *Prrx1^enh^-tdT-cKO* mice after receiving saline+vehicle (open circles), bleomycin+vehicle (open squares) and bleomycin+tamoxifen (filled squares). One-way ANOVA with Tukey's comparison test: *P≤0.05, **P≤0.01, ***P≤0.001. The absence of statistical labelling indicates non-significative results.

Lineage tracing revealed significant amplification of the *Prrx1^enh^*-positive population during the fibrotic phase following a single bleomycin insult (Fig. 2). To study the effect of *Prrx1* loss of function in this subpopulation during lung fibrosis, *Prrx1^enh^-tdT-cKO* animals underwent the post-bleomycin labelling protocol (Fig. 4A). To ensure that any potential worsening effect of *Prrx1* loss in this specific population would be properly assessed, a lower dose of bleomycin was used in this experimental setup (Fig. 4A). Consequently, the bleomycin+vehicle group exhibited only limited fibrotic lesions compared to the saline+vehicle-treated animals, as depicted in Figs 4 and 5. Furthermore, the loss of *Prrx1* in tdTomato-positive cells after tamoxifen treatment in *Prrx1^enh^-tdT-cKO* lungs, compared to *Prrx1^enh^-tdT* lungs, was confirmed by RNAscope *in situ* hybridization (Fig. S2).

Remarkably, we observed a pronounced increase in the extent of lung lesions at the histological level in the tamoxifen-treated group compared to the vehicle-treated group at Day 16 after bleomycin instillation (Fig. 4B,C). Additionally, we noticed a trend towards increased collagen deposition in the lungs of both bleomycin groups, particularly higher in the bleomycin+tamoxifen group, as assessed by Picrosirius Red staining intensity (Fig. 4C).

Furthermore, there was an increase in the number of cells in the BAL of both bleomycin mouse groups, regardless of tamoxifen treatment, compared to the saline+vehicle group (Fig. 4D). Notably, there was no significant difference between the two bleomycin groups. However, the tamoxifen-treated group exhibited a higher number of lymphocytes and polynuclear cells than did the vehicle-treated control group, which aligns with the increased lung damage observed in the bleomycin+tamoxifen group (Fig. 4D).

In addition to the upregulation of typical fibrosis-associated genes (*Col1a1*, *Fn1* and *Acta2*), an increase in *Prrx1a* and *Prrx1b* mRNA expression was previously reported during fibrosis development in the bleomycin model of lung fibrosis (Marchal-Duval et al., 2023). In the *Prrx1^enh^-tdT-cKO* animal model, the median mRNA expression level of all these genes was increased in both bleomycin-treated groups (tamoxifen or vehicle treated) compared to that in the saline+vehicle group (Fig. 4E).

These results showed that the loss of *Prrx1* in the *Prrx1^enh^*-positive subpopulation paradoxically upregulated the expression of *Prrx1* in the lung. This increase in *Prrx1* mRNA levels could have occurred through the *Prrx1*-positive population that did not activate the *Prrx1^enh^* cassette after bleomycin challenge. Notably, this increase was significantly higher for *Prrx1b* and *Col1a1* in the bleomycin+tamoxifen group compared to the bleomycin+vehicle group. These findings were confirmed at the protein level, with a significant increase in COL1, PRRX1 and FN1 protein levels in the bleomycin+tamoxifen group compared to those in the bleomycin+vehicle group (Fig. 4F,G). The fibrosis worsening observed in the lungs of *Prrx1^enh^-tdT-cKO* triple transgenic mice was not attributed to tamoxifen treatment. Indeed, this deterioration was not evident in the lungs of *Prrx1^enh^-tdT* double transgenic mice treated with tamoxifen or vehicle and subjected to a similar bleomycin challenge (M.H.-L., data not shown).

To further investigate profibrotic marker levels, we examined the expression of other fibrosis-associated genes (Fig. 5). *Prrx1* loss in the *Prrx1^enh^* subpopulation was associated with an increase in *Col3a1*, *Col14a1*, *Eln*, *Pai1* and *Ctgf* mRNA levels after injury in the bleomycin+tamoxifen group compared to those in the saline+vehicle group only (Fig. 5A,B). However, we did not find any significant difference or trend in the mRNA level of *Tnc* in vehicle-treated compared to tamoxifen-treated lung (Fig. S4). In contrast, regarding antifibrotic markers, we found a significant increase in *Hgf* and *Fgf10* mRNA levels in the bleomycin+tamoxifen group compared to those in both the saline+vehicle and bleomycin+vehicle groups (Fig. 5C). Moreover, although we did not observe a significant increase in *Fgf7* mRNA levels (Fig. 5C) in vehicle-treated compared to tamoxifen-treated lung (Fig. S3), we noticed a trend toward an increase in the tamoxifen-treated group. Interestingly, we detected an increase in the mRNA expression of profibrotic fibroblast subpopulation markers (Tsukui et al., 2020; Xie et al., 2018) – such as *Pdgfra*, *Pdgfrb*, *Cthrc1* and *Ebf1* – in the bleomycin+tamoxifen group compared to that in the bleomycin+vehicle group (Fig. 5D). Recent studies underscored the potential immunomodulatory function of the *Prrx1*-positive fibroblast population through the expression of key cytokines such as CCL2, CCL11, CCL7 (Ko et al., 2022, 2023) or CXCL12 (Lee et al., 2022). The fibrosis worsening observed in the *Prrx1^enh^-tdT-cKO* mice treated with bleomycin and tamoxifen was associated with significant increase in the mRNA expression of *Ccl7* or *Cxcl12* cytokines only, compared to that in the groups treated with saline and vehicle or bleomycin and vehicle (Fig. 5E).

**Fig. 6.**
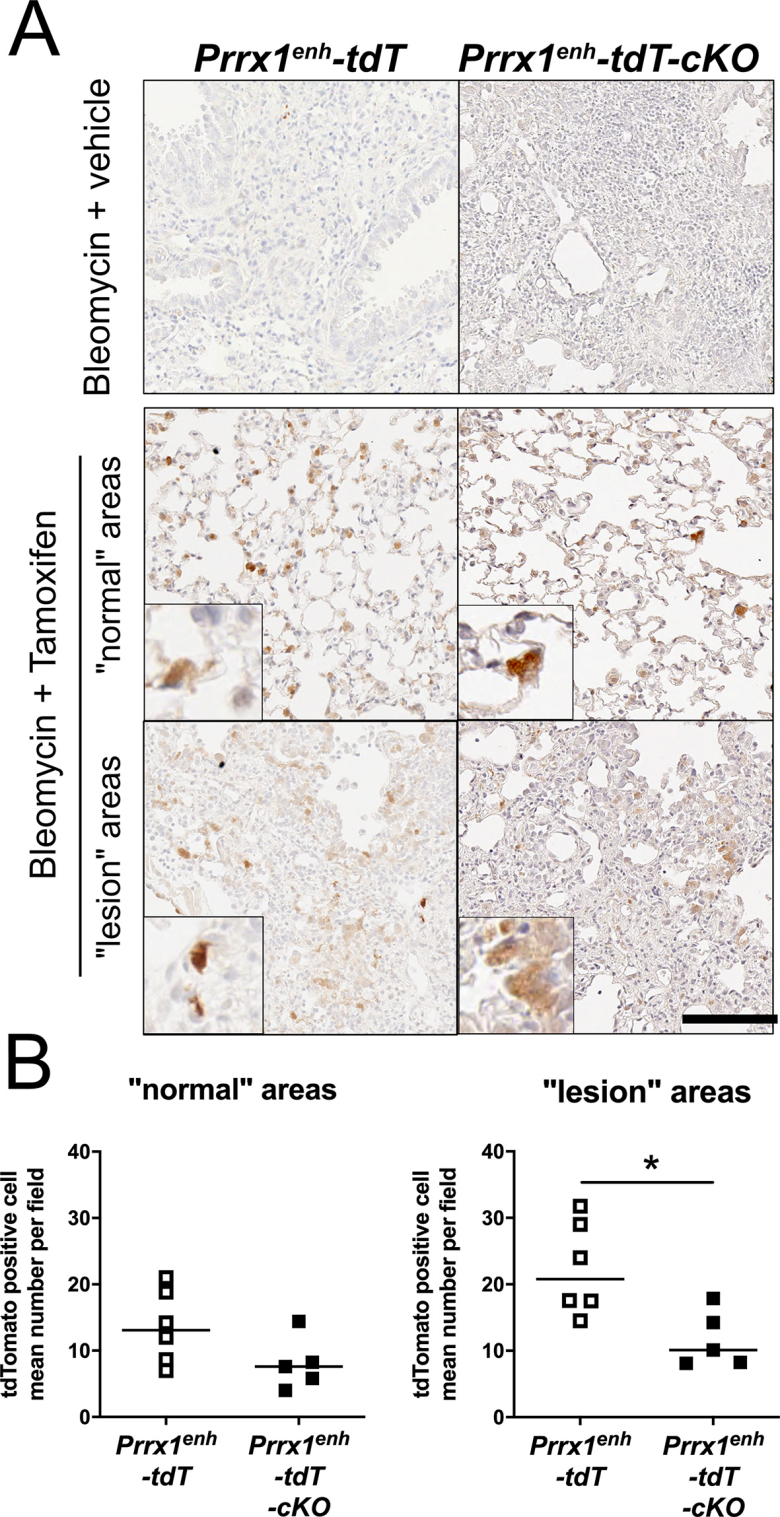
***Prrx1* loss of function in *Prrx1^enh^*-positive cells impacts their accumulation during bleomycin-induced lung fibrosis.** (A) Representative tdTomato immunohistochemistry in *Prrx1^enh^-tdT* (*n*=6) or *Prrx1^enh^-tdT-cKO* (*n*=5) mouse fibrotic lungs – normal and lesion zones – of the bleomycin+tamoxifen group (*n*=5)*.* Scale bar: 50 µm. (B) Quantification of tdTomato-positive cells in normal or lesion lung areas from the bleomycin+tamoxifen groups of *Prrx1^enh^-tdT* (open squares; *n*=6) and *Prrx1^enh^-tdT-cKO* (filled squares; *n*=5) mice; data shown as dot plots with median. Note that the *Prrx1^enh^-tdT* group data are reused from Fig. 2C,D (post-label group) to compare these data with the *Prrx1^enh^-tdT-cKO* animals. Mann–Whitney U-test: *P≤0.05. The absence of statistical labelling indicates non-significative results.

Finally, we sought to assess the potential impact of *Prrx1* deletion on the number of *Prrx1^enh^*-positive cells during fibrosis. To do so, we performed tdTomato IHC on the fibrotic lungs from *Prrx1^enh^-tdT-cKO* mice (Fig. 6A,B). Strikingly, we observed a significant reduction of ∼50% of the number of tdTomato-positive cells within the fibrotic zones of the lungs of *Prrx1^enh^-tdT-cKO* mice compared to that in the lungs of *Prrx1^enh^-tdT* lineage control mice (Fig. 6B). In addition, the two populations were not intermingled within the remodelled lung areas, maintaining a mean distance of 41 μm in post-labelled bleomycin-treated *Prrx1:Cre^ERT2^; Prrx1^fl/fl^; Rosa26iTomato* animals (Fig. S5)

In conclusion, our study strongly indicates that *Prrx1* loss of function in *Prrx1^enh^*-positive cells exacerbates bleomycin-induced lung fibrosis while impacting the accumulation of this specific cell population within the fibrotic lung.

## DISCUSSION

### Reactivation of developmental pathways and TFs during IPF

IPF is characterized by the reactivation of developmental pathways (SHH, Wnt, Notch and TGF-β) and downstream TFs [ETV (ETV4/5), β-catenin, GLI and SMADs, respectively] (Chilosi et al., 2003; Cigna et al., 2012; Froidure et al., 2020; Zhang et al., 2017; Zhao et al., 2002). Lung fibrogenesis was also associated with the aberrant activation of other TFs, such as YAP and TAZ (Gokey et al., 2018), Forkhead [FOXF1 (Pradère et al., 2014) and FOXO3 (Al-Tamari et al., 2018)], TBX [TBX4/5 (Horie et al., 2018)] and WT1 (Karki et al., 2014). More recently, the mesenchymal TF PRRX1 was found to be reactivated during IPF, expressed in fibroblast nuclei accumulating in fibroblastic foci (Marchal-Duval et al., 2023; Yeo et al., 2018). PRRX1 is one of the few TFs [with TBX4 (Horie et al., 2018)] to be restricted to the fibroblast/mesenchymal lineages in the lung (Marchal-Duval et al., 2023).

Recent single-cell RNA-sequencing analysis of the mouse lung has also shed light on the association between *Prrx1* expression and different fibroblast subpopulations. Notably, *Prrx1* is predominantly found in two distinct groups in the normal lung: the *Pi16*-positive adventitial fibroblasts and the *Col15a1*-positive parenchymal fibroblasts (Buechler et al., 2021; Marchal-Duval et al., 2023).

What is particularly intriguing is the discovery of *Prrx1* in the fibrosis-associated *Lrrc15*/*Cthrc1*-positive fibroblast population, which exhibits the highest levels of ECM protein production in animals treated with bleomycin (Marchal-Duval et al., 2023; Tsukui et al., 2020). Moreover, another single-cell analysis showed *Prrx1* expression in a *Col14a1*-positive matrix fibroblast cluster, which was exclusively activated in bleomycin-induced mouse fibrotic lungs (Xie et al., 2018). The significance of *Prrx1* in fibrosis has been further underscored by *in vivo* studies. We previously showed that inhibiting *Prrx1* using an ASO effectively attenuated bleomycin-induced lung fibrosis (Marchal-Duval et al., 2023).

The primary aim of the study was to gain a deeper understanding of the fate of *Prrx1-*positive mesenchymal cells in both normal and fibrotic mouse lungs, achieved through lineage tracing approaches utilizing the *Prrx1^enh^* promoter as Cre driver.

### Identification of a mesenchymal *Prrx1^enh^* subpopulation detectable only during lung fibrosis

Initially, only one promoter was available for performing *Prrx1* conditional lineage tracing in mice. It is composed of a 2.4 kb *Prrx1* minimal promoter sequence (*Prrx1^enh^*) found in the 5′ region of the *Prrx1* promoter, containing a 530 bp core of conserved regulating elements (Martin and Olson, 2000). This cassette, also named *Prrx1^enh^*, was initially used to generate a tamoxifen-inducible *Prx1CreER-GFP/Rosa26 LacZ* transgenic mouse line in which positive mesenchymal cells were labelled in the limbs and later in osteochondrocyte progenitors in mouse embryos (Kawanami et al., 2009). In the present study, we used this Cre driver to generate a *Prrx1:Cre^ERT2^; Rosa26iTomato* transgenic mouse line, to label *Prrx1*-expressing cells with tdTomato fluorescent protein after Cre-mediated recombination following a 5-day tamoxifen treatment. Although the activity of the *Prrx1^enh^* transgene in the ventral skin fibroblasts (Currie et al., 2019) and oral mucosa (Ko et al., 2023) in adult mice has been reported, its expression in the adult lung was not previously characterized. tdTomato IHC did not reveal any tdTomato-positive cells in the normal lung at baseline compared to the numerous positive cells detected in the ventral skin. PRRX1-positive cells are usually present in the perivascular and peribronchiolar spaces in the normal lung (Marchal-Duval et al., 2023).

These results suggest that the *Prrx1^enh^* promoter is sufficient to target the majority of *Prrx1*-positive cells in the limb and ventral skin. However, it appears to be either inactive in the lung or labels only a very rare *Prrx1^enh^*-positive subpopulation within the broader *Prrx1*-positive lung cell pool – one that is challenging to detect by IHC in FFPE lung sections in healthy adult mice. A more sensitive method, such as fluorescence-activated cell sorting, could potentially enable the identification of this rare population. Nevertheless, the known leakiness of the tdTomato reporter allele in the Ai9 mouse line (Homps-Legrand et al., 2023), as noted in the strain details provided by The Jackson Laboratory, limited our ability to distinguish true signal from background by flow cytometry analysis – even in bleomycin-treated animals. Future studies could benefit from using an alternative reporter line with minimal basal expression, such as the *R26R-EYFP* mouse line (Homps-Legrand et al., 2023), which may improve the detection of low-abundance cell populations. Similarly, the *Prrx1^enh^* promoter was shown to be active in only a subset of *Prrx1*-expressing mesenchymal cells in the oral mucosa (Ko et al., 2023), indicating that the *Prrx1^enh^* cassette does not drive expression in all *Prrx1*-positive cells within a given tissue. Recently, an alternative *Prrx1*-Cre driver line was developed, in which a *Cre^ERT2^* cassette was inserted directly into the *Prrx1* locus (Liu et al., 2022). This knock-in allele may offer a more physiologically relevant and comprehensive model for targeting *Prrx1*-expressing cells.

Next, we observed significant accumulation of tdTomato-positive cells in the lungs following bleomycin-induced lung fibrosis, predominantly concentrated in fibrotic areas. Notably, the count of tdTomato-positive cells was found to be ten times higher when the cells were labelled during the fibrotic phase (post-bleomycin labelling protocol), compared to labelling with tamoxifen before bleomycin injury (pre-bleomycin labelling protocol). These findings conclusively demonstrated the activation of the *Prrx1^enh^* promoter during lung fibrosis following bleomycin exposure. It is worth mentioning that a similar increase in the *Prrx1^enh^*-positive subpopulation was previously reported after dermal injury (Currie et al., 2019).

In the fibrotic lung, the tdTomato-expressing cells exhibited a CD45^neg^ CD31^neg^ VIM^pos^ PDGFRα^pos^ marker profile. This specific cell marker profile is typically associated with mesenchymal cells, particularly fibroblasts. In addition, these cells were negative for ACTA2 and CTHRC1 in the remodelled parenchyma, suggesting that *Prrx1^enh^* did not label myofibroblast progeny in this context. These results are consistent with the existing data that identifies *Prrx1* as mesenchymal TFs playing a crucial role in tissue fibrosis and repair (Currie et al., 2019; Jiang and Stefanovic, 2008; Ko et al., 2023; Leavitt et al., 2020; Marchal-Duval et al., 2023; Yeo et al., 2018).

### The *Prrx1^enh^* subpopulation displays antifibrotic properties in the lung

*PRRX1* has been recognized as a master TF of stromal fibroblasts, driving their differentiation into myofibroblasts (Lee et al., 2022). Therefore, we aimed to reprogram the *Prrx1^enh^*-positive subpopulation, following conditional and inducible *Prrx1* loss of function, utilizing a newly generated floxed *Prrx1* allele. To achieve this, we utilized the *Prrx1:Cre^ERT2^; Prrx1^fl/fl^; Rosa26iTomato* transgenic mouse line to investigate the functional role of the *Prrx1^enh^* subpopulation *in vivo*. Consistent with the lack of *Prrx1^enh^* transgene efficient activity in uninjured lungs, the analysis of the *Prrx1:Cre^ERT2^; Prrx1^fl/fl^; Rosa26iTomato* lungs at baseline after tamoxifen treatment revealed no significant effect on lung architecture or inflammation.

Building upon our lineage tracing findings, we proceeded to investigate the impact of *Prrx1* loss of function within the *Prrx1^enh^* subpopulation in the context of lung fibrosis. Upon analysis, the results unveiled an unexpected outcome, indicating that *Prrx1* loss of function in *Prrx1^enh^*-positive mesenchymal cells aggravated bleomycin-induced lung fibrosis *in vivo*. This aggravation was evident through an increase in both profibrotic markers and the activation of fibroblast subpopulation markers, which were exclusive to the fibrotic context. Intriguingly, our observations also revealed a paradoxical increase in repair-associated markers – namely, *Fgf7*, *Fgf10* and *Hgf* – following *Prrx1* loss of function within this specific subpopulation. This finding suggests that the absence of *Prrx1* in these cells initiates a defective repair feedback loop in the context of lung fibrosis. Indeed, despite the observed upregulation of those markers, we did not observe the presence of features typically associated with actual lung repair (Basil et al., 2020), such as epithelial pods or an increase in TTF1-positive epithelial cells in the lungs (M.H.-L., data not shown). Also, our findings revealed a significant decrease in the count of tdTomato-positive cells following *Prrx1* deletion within the *Prrx1^enh^* subpopulation in tamoxifen-treated *Prrx1:Cre^ERT2^; Prrx1^fl^; Rosa26iTomato* lungs during lung fibrosis. Considering the known involvement of PRRX1 in lung fibroblast proliferation (Marchal-Duval et al., 2023), the observed decrease in tdTomato-positive cells could be attributed to *Prrx1* loss within this *Prrx1^enh^* population in this context.

**Fig. 7.**
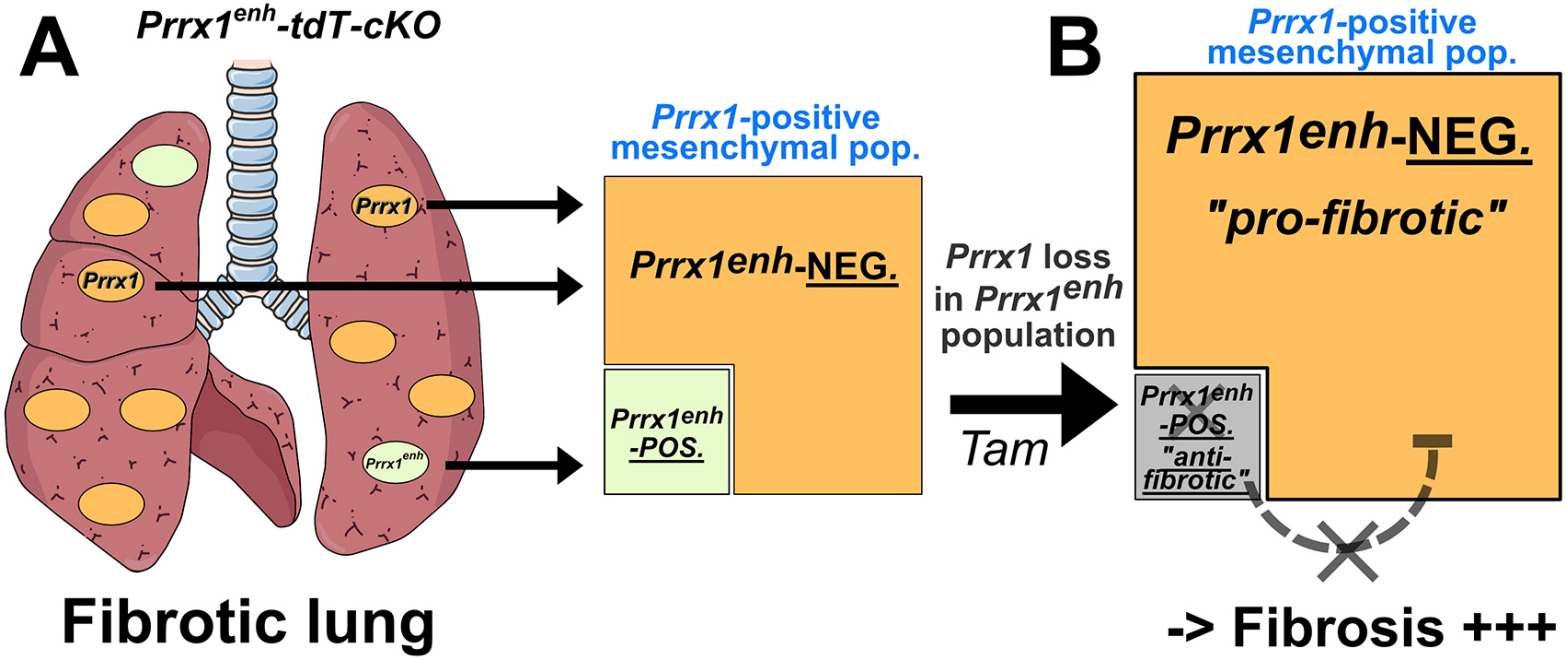
**Summary of the effects of PRRX1 loss in the *Prrx1^enh^*-positive population in the lungs of *Prrx1^enh^-tdT-cKO* animals after bleomycin treatment.** (A) After bleomycin challenge, two *Prrx1*-positive cell populations are accumulating in the lung, distinct in their activation status of the *Prrx1^enh^* cassette: a rare *Prrx1^enh^*-positive population (green square) and a prominent *Prrx1^enh^*-negative but *Prrx1*-positive population (orange polygon), as shown by the respective sizes of the coloured forms. (B) *Prrx1* loss in this sparse *Prrx1^enh^*-positive population using a *Prrx1* floxed allele (*Prrx1^enh^-tdT-cKO* transgenic animal after tamoxifen treatment) was associated with a decrease in this subpopulation (grey square), concomitantly with an increase in fibrosis and accumulation of the *Prrx1^enh^*-negative, but still *Prrx1*-positive, profibrotic population (orange polygon). These observations suggest that the *Prrx1^enh^*-positive cell subpopulation displays antifibrotic properties in the lung upon this experimental setup. NEG, negative; pop, population; POS, positive; *Prrx1^enh^-tdT-cKO*, *Prrx1:Cre^ERT2^; Rosa26iTomato; Prrx1^fl/fl^* mice; Tam, tamoxifen*.*

A paradoxical increase in the expression of *Prrx1* TFs was measured, suggesting the promotion of a *Prrx1*-positive, but *Prrx1*^enh^-negative, cell population. As PRRX1 is known as a fibroblast lung marker (Lee et al., 2022; Marchal-Duval et al., 2023; Yeo et al., 2018), these results imply that *Prrx1* loss of function in the *Prrx1^enh^*-positive population reduces this same population but led to the accumulation of other *Prrx1*-expressing mesenchymal cell populations (see Fig. 7). Notably, our findings potentially unveil the existence of two *Prrx1*-positive subpopulations: a rare *Prrx1^enh^* subpopulation exhibiting antifibrotic features and a more prominent population exhibiting profibrotic properties after lung injury. In fact, the potential effect of the *Prrx1^enh^* population appears to be context dependent, varying in different injury scenarios. In the mouse ventral dermis, the amplification of this *Prrx1^enh^* subpopulation was associated with fibrosis development after skin injury (Currie et al., 2019; Leavitt et al., 2020). Conversely, in the oral mucosa, the same *Prrx1^enh^* population led to better and faster regeneration (Ko et al., 2023). Additionally, in a pancreatic cancer mouse model, *Prrx1* conditional loss of function in cancer-associated fibroblasts, using a different *Prrx1^fl^* allele and the *Sm22-Cre^ERT^* driver mouse line, resulted in increased extracellular matrix deposition and the accumulation of myofibroblasts (Lee et al., 2022). A potential immunomodulatory effect could be associated with *Prrx1* loss in this population, as we observed an increase in neutrophil count in BAL and in mRNA lung levels of cytokines such as *Ccl7* or *Cxcl12*, in tamoxifen-treated animals compared to those in vehicle control ones after bleomycin challenge. These two chemokines are known to play a role in role in both recruitment and retention of neutrophils (Struyf et al., 2005). The immunomodulatory effect of the *Prrx1^enh^* subpopulation was underscored in the different studies mentioned above (Ko et al., 2022, 2023; Lee et al., 2022).

In our previous study, we reported an antifibrotic effect of global *Prrx1* inhibition using an ASO approach during the fibrotic phase after bleomycin challenge (Marchal-Duval et al., 2023). One should bear in mind that this experimental setup targeted both *Prrx1^enh^*-positive and *Prrx1^enh^*-negative cells within PRRX1-expressing mesenchymal populations during lung fibrosis. Hence, the inhibition of *Prrx1* in the profibrotic population showed more beneficial effects than did the loss of *Prrx1* in the *Prrx1^enh^* antifibrotic population.

In conclusion, our experiments have identified an antifibrotic *Prrx1^enh^* subpopulation among mesenchymal *Prrx1*-positive cells, rare in the normal lung but accumulating during fibrosis development. These findings shed light on the complex and unexpected role of *Prrx1* in modulating fibrosis progression, underscoring the importance of exploring specific subpopulations within the mesenchymal compartment to gain a comprehensive understanding of fibrotic processes.

## MATERIALS AND METHODS

### *In vivo* experiments

All mouse experiments were performed using males of C57BL/6J genetic background. The *B6.Cg-Tg(Prrx1-cre/ERT2,-EGFP)1Smkm/J* mouse line (Kawanami et al., 2009) – referred as *Prrx1:Cre^ERT2^* – was provided by Pr Murakami (Cleveland, OH, USA). The *B6.Cg-Gt(ROSA)26Sortm9(CAG-tdTomato)Hze/J* (*Ai9*) line – referred as Rosa26iTomato – was described elsewhere (Madisen et al., 2010). The *Prrx1^fl/fl^* mouse line was generated in collaboration with PHENOMIN – French National Infrastructure for Mouse Phenogenomics (Illkirch, France). The *Prrx1:Cre^ERT2^; Rosa26iTomato* animals used for lineage tracing are designated as *Prrx1^enh^-tdT* in the article*.* The *Prrx1:Cre^ERT2^; Prrx1^fl/fl^; Rosa26iTomato* mice used for lineage tracing and *Prrx1* conditional knockout (cKO) are designated as *Prrx1^enh^-tdT-cKO* in the article. Genotyping primers are listed in Table S1.

### *In vivo* experiments

In all protocols, mice were intratracheally instilled with 50 µl bleomycin [Bellon, lower dosage of 30 µg per mouse instead of 50 μg (Moshai et al., 2014)] or saline as described elsewhere (Jenkins et al., 2017; Moeller et al., 2008). Tamoxifen (Sigma-Aldrich) was dissolved in 100% ethanol (100 mg/ml) after sonication, and then in corn oil (vehicle, 10 mg/ml), as previously described (Bordenave et al., 2020).

Two protocols were performed with respect to lung fibrosis after bleomycin challenge. In protocol #1 (pre-bleomycin labelling), tamoxifen or vehicle was intraperitoneally injected to mice from Day −10 to Day −5, and bleomycin was instilled at Day 0. In protocol #2 (post-bleomycin protocol), bleomycin was first instilled at Day 0, and then tamoxifen or vehicle only was injected from Day 7 to Day 11. Lungs were harvested on Day 14 post-bleomycin in protocol #1 and at Day 16 in protocol #2 (after a 5-day wash-out after tamoxifen treatment in the second protocol). BAL was performed as described previously (Phin et al., 2010). Haematoxylin, Eosin and Picrosirius Red staining was performed routinely to evaluate the lung morphology. Semiquantitative histological assessment of lung injury used the grading system described by Inoshima and colleagues (Inoshima et al., 2004). Total mRNA was extracted from mouse lung homogenates, and expression of the genes of interest was quantified by real-time quantitative PCR (qPCR), as previously described. Proteins were extracted from mouse lung homogenates, and western blotting was performed by standard techniques as previously described (Moshai et al., 2014).

### MEFs

MEFs were prepared and immortalized from embryonic day 13.5 *Prrx1^fl/fl^; Rosa26iTomato* mouse embryos and cultured as described elsewhere (Xu, 2005). MEFs were cultured with Dulbecco's modified Eagle medium (Thermo Fisher Scientific) supplemented with 10% fetal calf serum and antibiotics. *Prrx1^fl/fl^; Rosa26iTomato* MEFs were then infected with adenoviral vector encoding Cre recombinase (Had5-Cre, 5.10^7^ plaque-forming units, provided by Plateforme de Vectorologie de Montpellier (Plateau IGMM, Montpellier, France) to trigger the excision of the sequences of interest surrounded by *LoxP* sites, leading to *Prrx1* loss of function and tdTomato gain of expression. The tdTomato-positive cells were subcloned for further analysis.

Genotyping was performed with primers located around *LoxP* sequences to detect Cre recombinase-mediated exon 2 excision in the *Prrx1* locus. Briefly, genomic DNA was extracted by successive centrifugations of acetic acid, 100% ethanol and 70% ethanol. The primers used for *Prrx1^fl^* allele genotyping are listed in Table S1. The *Prrx1^fl^* conditional allele is measured at 1066 bp, and the *Prrx1* knock-out allele – obtained after deletion of exon 2 – is measured at 239 bp, on a 2% agarose gel.

Immunofluorescence was performed on MEFs cultured in complete growth medium on Permanox Lab-Tek Chamber Slides (Nunc, Thermo Fisher Scientific), after paraformaldehyde fixation and 0.25% Triton X-100 permeabilization, using an anti-RFP antibody (with a donkey anti-rabbit AF568 secondary antibody) and an anti-PRRX1 antibody (with a donkey anti-mouse AF488 secondary antibody) (see Table S2). Nuclei were stained with 4',6-diamidino-2-phenylindole (DAPI).

### Reverse transcription qPCR

Total mRNA was extracted from mouse lung homogenates (Ambion). Retro-transcription into cDNA was performed with 500 ng total mRNA (Moshai et al., 2014). *Rn18s* (shown as ‘*18S*’ in figures) was used as housekeeping gene for qPCR. The expression of *Prrx1a*, *Prrx1b*, *Col1a1*, *Fn1*, *Acta2*, *Col3a1*, *Col14a1*, *Eln*, *Tnc*, *Ctgf*, *Tgfb1*, *Pai1*, *Fgf7*, *Hgf*, *Fgf10*, *Pdgfra*, *Pdgfrb*, *Cthrc1*, *Ebf1*, *Ccl2*, *Ccl11*, *Ccl7*, *Cxcl12* and *Rn18s* mRNA was quantified by real-time PCR (see Table S1 for primers) with the use of PCR QuantStudio 6 (Applied Biosystems).

### Western blotting

Total proteins were extracted with RIPA buffer (10% Triton X-100, 5% sodium deoxycholate, 20% SDS, 2 M Tris-HCl pH7.5, 0.5 M NaCl, 0.25% EDTA) and anti-proteases. Proteins were dosed by Bradford method, and 40 µg per well was run on 4-15% gradient criterion TGX gels (2553071, Bio-Rad). Protein transfer was performed on PVDF membranes (Moshai et al., 2014). HSC70 or TUB were used as loading controls. Anti-PRRX1, anti-COL1, anti-FN1, anti-ACTA2, anti-TUB and anti-HSC70 antibodies were diluted in TBS-0.1% Tween 20-5% milk (see Table S2). Images were obtained with a PXi4 camera (Syngene).

### Histology, IHC and RNAscope analysis

FFPE sections were treated for single and double IHC labelling as previously described in Joannes et al. (2016) and Marchal-Duval et al. (2023). Antibodies are reported in Table S2. To validate the specificity of immunostaining, antibodies were replaced by a matched control isotype. All digital images of light microscopy were acquired with a DM400B microscope (Leica) equipped with a Leica DFC420 CDD camera and analysed with Calopix software (TRIBVN). With respect to tdTomato-positive cell quantifications, ‘normal’- versus ‘lesion’-looking areas were chosen based on the lack or presence, respectively, of major alveolar thickening as well as fibrous changes and masses (confirmed by Picrosirius Red staining on serial sections). The distance between tdTomato-positive cells and PRRX1-positive ones was measured with ImageJ, using the tdTomato-positive cells and the nearest PRRX1-positive cells in lesion-looking areas as reference (at least seven pictures at 20× magnification per lungs were analysed).

Double multiplex immunofluorescence was performed as previously described (Chen et al., 2010) using TSA secondary antibody amplification kits (Tyramide SuperBoost Kit, B40944 and B40941, Invitrogen). Briefly, tdTomato immunofluorescence was first performed. Sections were then incubated after citrate heat-induced epitope retrieval with anti-vimentin, anti-PDGFRα, anti-ACTA2 (αSMA), anti-NG2, anti-CC10, anti-CD45, anti-CTHRC1 or anti-CD31 antibodies (see Table S2). Lung sections were stained with DAPI for nuclei visualization. Quenching was performed to reduce autofluorescence (Vector TrueBlack, SP-8400, Vector Laboratories). Images were obtained using a Leica SP8 confocal microscope (original magnification 63×).

RNAscope *in situ* hybridization was performed using an RNAscope 2.5 HD Duplex Detection Reagent kit (322500) on paraffin-embedded lung sections following the manufacturer's (Advanced Cell Diagnostics) instructions. The following probes were used: Mm-Prrx1 (485231) and tdTomato-C2 (31704-C2). The specificity of the probes was validated on paraffin sections of *Prrx1^fl/fl;^ Rosa26iTomato* MEF pellets with or without infection with adenoviral vector encoding Cre recombinase (M.H.-L., data not shown).

### Statistical analysis

Data are represented as dot plots with median, unless specified. All statistical analysis were performed using Prism 6 (GraphPad Software). We used non-parametric Mann–Whitney *U*-test for comparison between two experimental conditions. One-way ANOVA with Tukey’s comparison test was used when at least three experimental conditions were compared. Comparison of histological scores and BAL cell types on Day 16 was performed with chi-square exact test. *P*<0.05 was considered statistically significant. Exact *P*-values and definitions and numbers of replicates are provided in the figure legends.

### Study approval

All animal experiments were conducted in accordance with the Directive 2010/63/EU and approved by the local animal ethics committee (Comité d'éthique Paris Nord n°121, APAFiS #4778 Etudedufacteurdetran_2016031617411315).

## Supplementary Material

10.1242/dmm.052179_sup1 Supplementary information
