# Supplementary material for: Uncovering an antifibrotic Prrx1-lineage mesenchymal cell subpopulation in fibrotic lungs
Source: Dis Model Mech. 2025 Aug 26;18(8):dmm052179. doi: 10.1242/dmm.052179 (PMC12421803; doi:10.1242/dmm.052179)
Supplement: Supplementary information [file dmm-18-052179-s1.pdf]

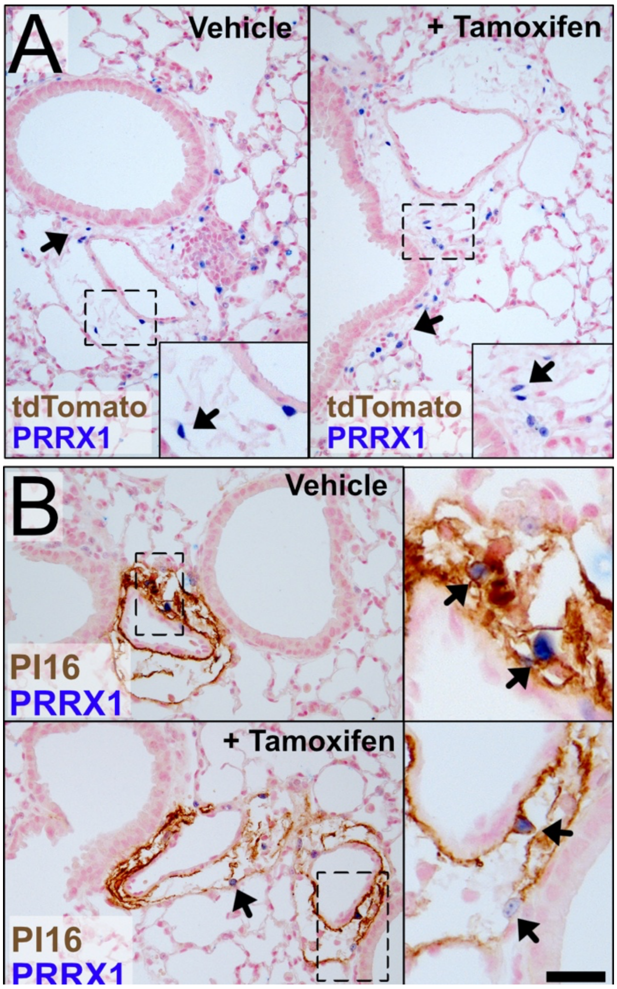

Fig. S1. Efficiency of labelling of PRRX1^{pos} cells in the lung of untreated *Prrx1:Cre^{ERT2}; Rosa26iTomato* animals.

(**A**) Representative microphotograph of double immunohistochemistry for PRRX1 (Blue chromogen) and tdTomato (Brown chromogen) *in Prrx1:Cre^{ERT2}; Rosa26iTomato* (*Prrx1^{enh}-tdT-cKO*) animals with vehicle (left panel) or tamoxifen (right panel). Note that the PRRX1 -positive cells were not labelled (tdTomato negative – black arrows). The inserts showed the area in dashed boxes. Slides were counterstained with nuclear Fast Red (**B**) Representative microphotograph of double immunohistochemistry for PRRX1 (Blue chromogen) and PI16 (Brown chromogen) *in Prrx1:Cre^{ERT2}; Rosa26iTomato* (*Prrx1^{enh}-tdT-cKO*) animals with vehicle (upper panels) or tamoxifen (lower panels). Slides were counterstained with nuclear Fast Red Note that the PRRX1 positive cells were PI16 positive (arrows) identifying them as adventitial fibroblasts. The right panels showed the area in dashed boxes in left panels. Scale bar: 80µm (main panels in A); 25µm (inserts in A); 40µm (left panels in B), 15µm (right panesl in B).

Disease Models & Mechanisms • Supplementary information

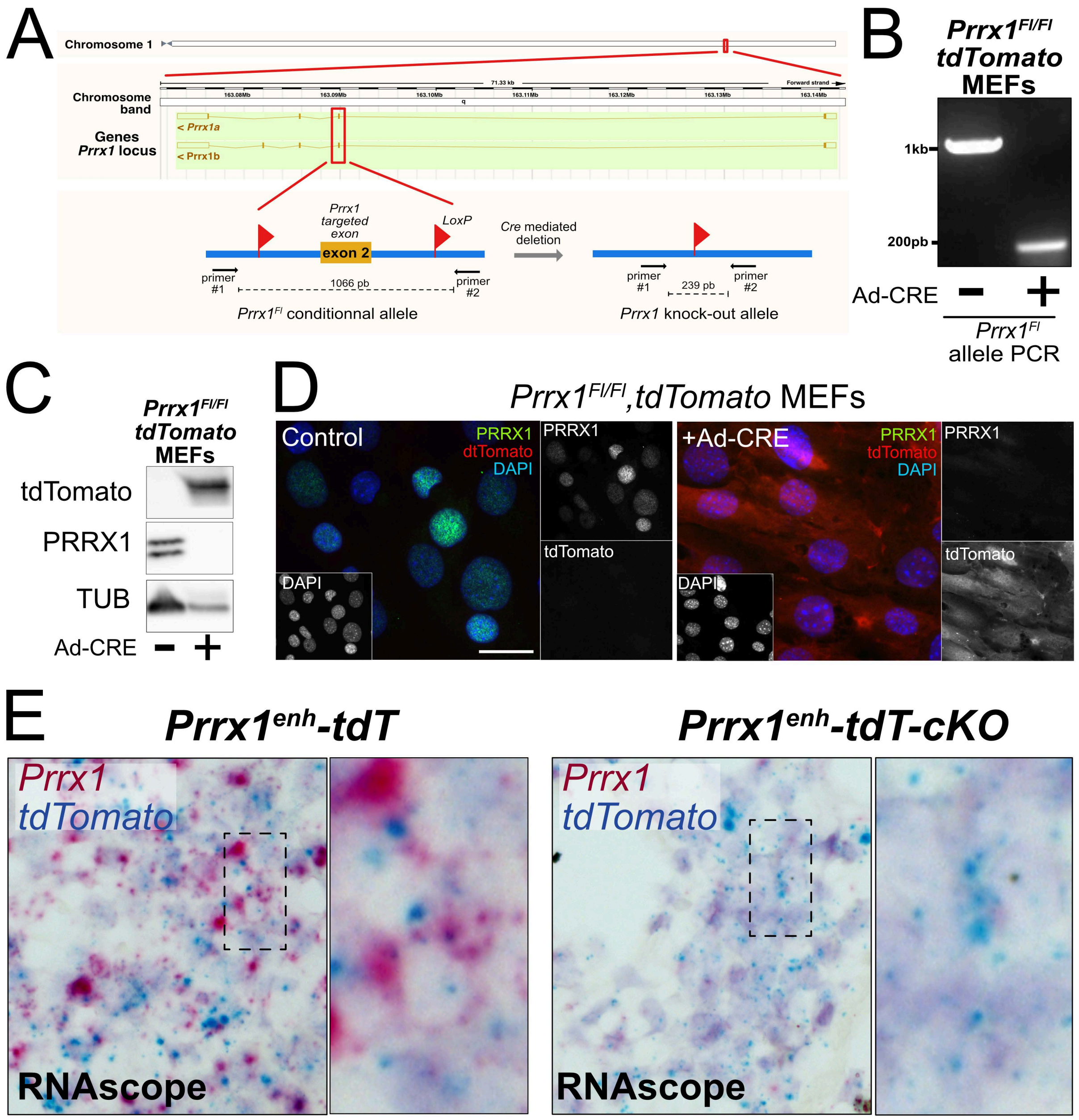

Fig. S2. Generation and validation of the *Prrx1^Fl^* allele.

(**A**) Schematic description of the *Prrx1^Fl^* allele targeting the exon 2 of both *Prrx1a* and *Prrx1b* genes. Genotyping is performed using primers located around the *LoxP* sites: *Prrx1^Fl^* conditional allele allows the amplification of a 1066 bp DNA sequence, while *Prrx1* knock-out allele – obtained after CRE-mediated deletion – only amplifies a 239 bp sequence. (**B**) Genotyping PCR of the *Prrx1^Fl^* allele of *Prrx1^Fl/Fl^; Rosa26iTomato* murine embryonic fibroblasts (MEFs) treated or not with a CRE adenovirus (Ad-CRE), according to the scheme of the *Prrx1^Fl^* allele. (**C**) Immunoblot showing tdTomato and PRRX1 expression in *Prrx1^Fl/Fl^; Rosa26iTomato* murine embryonic fibroblasts (MEFs) treated or not with a CRE adenovirus. TUB is used as loading control. Note the presence of tdTomato protein and PRRX1 loss in Ad-CRE transduced MEFs. (**D**) Immunofluorescence showing PRRX1 (green) and tdTomato (red) expression in control (left panel) or Ad-Cre (right panel) transduced *Prrx1^Fl/Fl^; Rosa26iTomato* murine

Disease Models & Mechanisms • Supplementary information

embryonic fibroblasts (MEFs). Separate channels in grey scale are presented on the right of each image. DAPI (blue) was used for nuclei staining, and its separate channel is presented on the bottom left of each image. Note the absence of PRRX1 nuclear staining in the tdTomato-positive MEFs transduced with CRE adenovirus (left panel) while control MEFs were PRRX1-positive and tdTomato-negative (right panel). (**E**) RNAscope in situ hybridization in *Prrx1^{enh}-tdT* (left panel) or *Prrx1^{enh}-tdT-cKO* (right panel) lung sections ("post-bleomycin" labelling protocol) to detect *Prrx1* mRNA (Red Chromogen) and *tdTomato* mRNA (Cyan chromogen) - representative pictures (n=3 per group). Note the accumulation of *Prrx1*-negative but *tdTomato*-positive cells in the fibrotic areas of *Prrx1^{enh}-tdT-cKO* lung samples compared to control *Prrx1^{enh}*-tdT lung ones, which display numerous *Prrx1*-positive cells. The regions at high magnification on the right of each panel correspond to those delineated by dashed rectangles. Scale bar: 6µm in D; 50µm in main panels and 15µm in high magnifications in E. *Abbreviations: murine embryonic fibroblasts (MEFs), Flox (Fl), CRE adenovirus (Ad-CRE).*

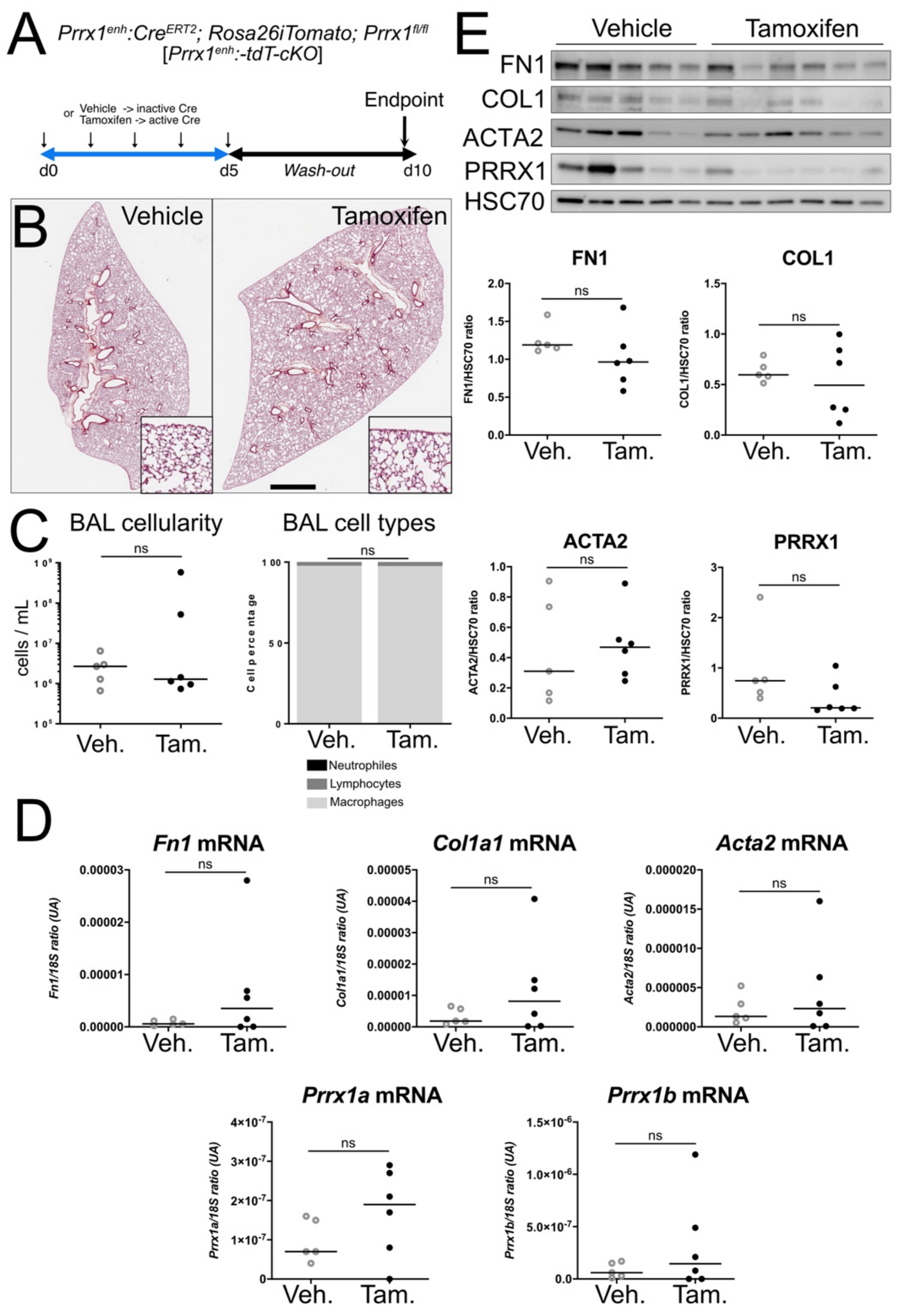

Fig. S3. *Prrx1* loss of function in *Prrx1*^{enh}-positive cells showed a slight lung impact at baseline.

(**A**) Timeline of vehicle and tamoxifen treatments. *Prrx1*^{enh}*-tdT-cKO* mice were injected with vehicle or tamoxifen every day for five days. Lungs were collected at Day 10 after a wash-out time of 5 days. (**B**) Representative pictures (n=5 in vehicle group and n=6 in tamoxifen group) showing Hematoxylin eosin staining in *Prrx1*^{enh}*-tdT-cKO* mouse lungs after 5 days of tamoxifen or vehicle treatment followed by a 5-day wash-out. High magnification pictures are displayed in the main panels. (**C**) BAL cell count (left part) in vehicle (white circle) and tamoxifen (black circle) groups. Right panel: quantification of BAL cell types – polynuclear cells (black),

Disease Models & Mechanisms • Supplementary information

lymphocytes (dark grey) and macrophages (light grey) – in vehicle (n=5) and tamoxifen (n=6) groups. (**D**) Dot plots with median showing the mRNA expression of *Prrx1a*, *Prrx1b*, *Col1a1*, *Fn1* and *Acta2* in *Prrx1^{enh}-tdT-cKO* mouse lungs after 5 days of vehicle (white circle, n=5) or tamoxifen (black circle, n=6) treatment followed by a 5-day wash-out. (**E**) Immunoblot showing PRRX1, COL1, FN1 and ACTA2 expression in mouse lungs after 5 days of vehicle or tamoxifen treatment followed by a 5-day wash-out. HSC70 was used as loading control. The quantification of PRRX1, COL1, FN1 and ACTA2 relative expression to HSC70 of vehicle (white circle, n=5) and tamoxifen (black circle, n=6) groups is displayed as dot plots with median on the lower panel. Scale bar: 100µm in main panels and 25µm in high magnifications. *Mann-Whitney U test, ns non-significative, *p≤0.05.*

Disease Models & Mechanisms • Supplementary information

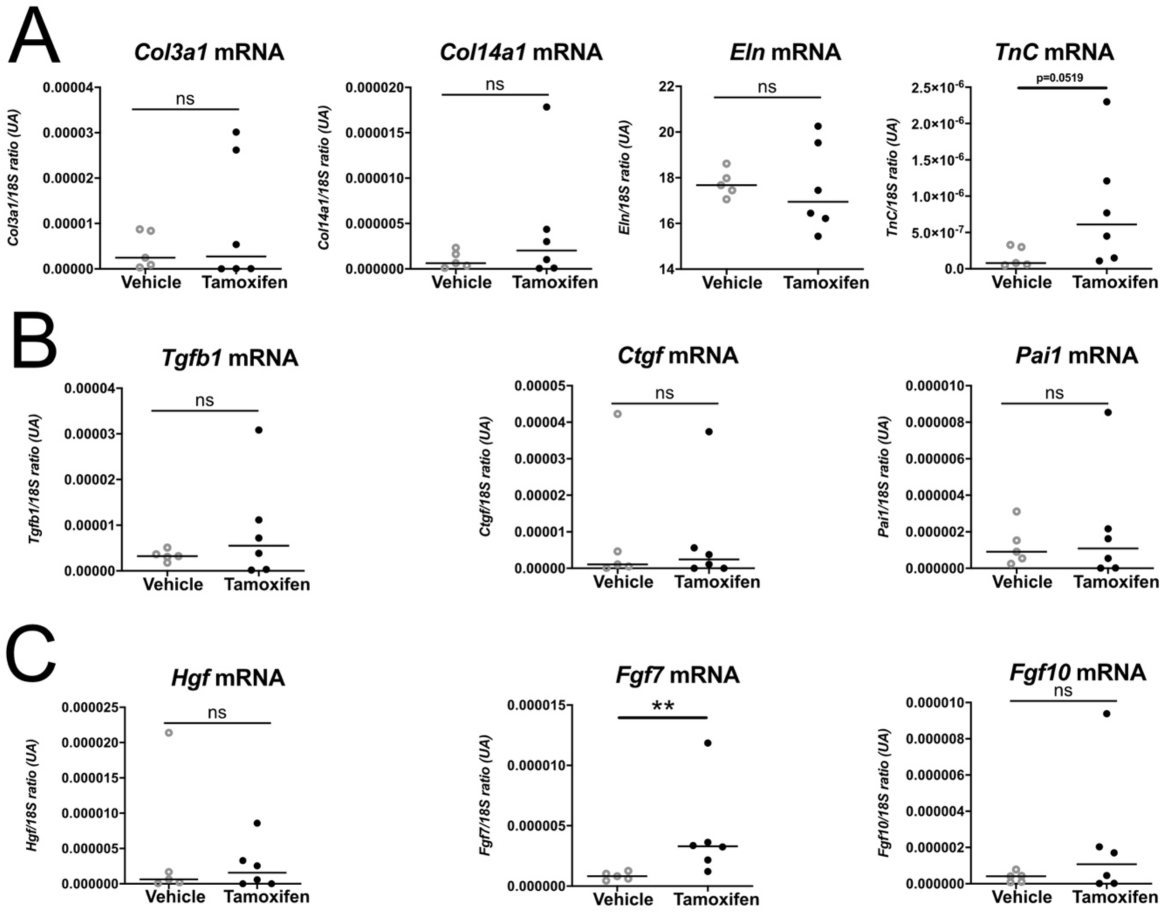

Fig. S4. mRNA expression of lung fibrosis associated markers after *Prrx1* loss in *Prrx1*^{enh}-positive cells at baseline.

(**A-C**) Dot plots with median showing the mRNA expression of (A) the profibrotic markers *Col3a1*, *Col14a1*; (B) *Tnc*, *Eln*, *Tgfb1*, *Pai1* and *Ctgf* and (C) the antifibrotic markers *Hgf*, *Fgf7* and *Fgf10* in *Prrx1*^{Enh}*-tdT-cKO* mouse lungs after 5 days of vehicle (white circle, n=5) or tamoxifen (black circle, n=6) treatment followed by a 5-day wash-out. *Mann-Whitney U test, ns non-significative, **p≤0.01.*

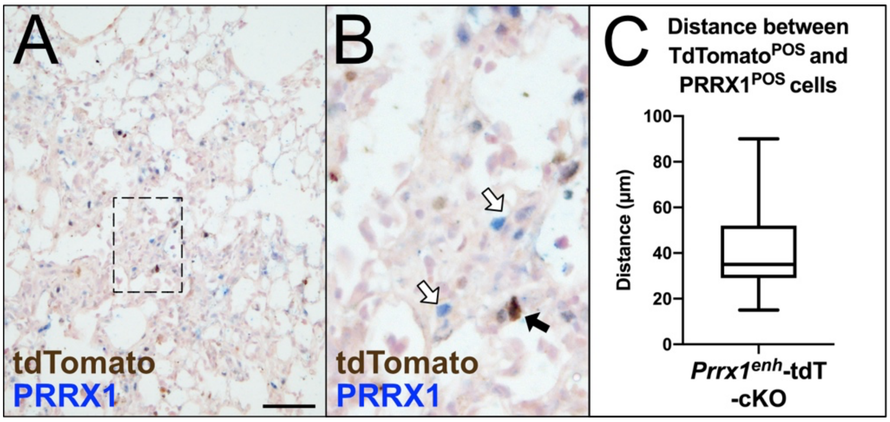

Fig. S5. *Prrx1^{enh}*-expressing cells are not in direct contact with PRRX1-positve *Prrx1^{enh}*-negative cells in the lung of bleomycin treated *Prrx1:Cre^{ERT2}; Prrx1$^{Fl/Fl}$;Rosa26iTomato* mice.

(**A**) Representative microphotograph of double immunohistochemistry for PRRX1 (Blue chromogen) and tdTomato (Brown chromogen) *in Prrx1:Cre^{ERT2}; Prrx1$^{Fl/Fl}$; Rosa26iTomato* (*Prrx1^{enh}-tdT-cKO*) animals treated with bleomycin and tamoxifen (post bleomycin labelling protocol -see Figure 4A). Nuclei were counterstained with nuclear fast red. (**B**) High magnification of the dashed area in (A) showing tdTomato-positive cells (Brown, black arrow) and PRRX1-positive cells (Blue, white arrows). (**C**) Box diagram showing the distance between tdTomato-positive cells and the nearest PRRX1-positive cells (n=4 *Prrx1:Cre^{ERT2}; Prrx1$^{Fl/Fl}$; Rosa26iTomato* animals). Scale bar : 50µm (A) and 20µm (B).

Table S1. Primers used for qPCR and genotyping PCR.

Gene	Forward Primer	Reverse Primer
Prrx1a	CTCTCCGTACAGCGCCAT	GTTGGCCATGTTGATACCCT
Prrx1b	CCGTACAGATCTTCGTCCCT	TTCCTCAGTTGACTGTTGGC
Col1a1	GTGTGTGACAAGGGTGAGACA	GAGAACCAGGAGAACCAGGA
Fn1	TGGTGGCCACTAAATACGAA	GGAGGGCTAACATTCTCCAG
Acta2	AGTCGCTGTCAGGAACCCTGAGA	ATTGTCGCACACCAGGGCTGTG
Rna18S	CTTAGAGGGACAAGTGGCG	ACGCTGAGCCAGTCAGTGTA
Prrx1^{Fl} allele	CCCGTCTTTGGTGAAATGCAGAGAAC	GCTCAGGTGAGGAAGAGTTATCTATGGCC
Col3a1	TACACCTGCTCCTGTGCTTC	CATTCCTCCCACTCCAGACT
Col14a1	GTTCAACGTGGGCTCAGAAA	ACTCCTCGATCCTGCTTCTG
Eln	GTTGGCGGAGTCCCAGGT	AGCACCTGTCGAGACTCCTA
Tnc	GGCGTCCCATTACAGAGGAA	CCGTGAAGAAGTACCTGGGT
Ctgf	*TGGGAGAACTGTGTACGGAG*	*GCTGCTTTGGAAGGACTCAC*
TgfB	*CTTCAGCTCCACAGAGAAGAACTGC*	*CACAATCATGTTGGACAACTGCTCC*
Pai1	*CCATGATGGCTCAGAACAAC*	*GTAGGGCAGTTCCAGGATGT*
Fgf7	*CACCTCGTCTGTCTAGTGGG*	*GTCCCTTTCACTTTGCCTCG*
Hgf	*CGTAGCGTACCTCTGGATTGC*	*ACCAGTAGCATCGTTTTCTTGA*
Fgf10	*AGGCTGTTCTCCTTCACCAA*	*TCCCCTTCTTGTTCATGGCT*
Pdgfra	*GTCTCAGGAGCTATGGGGAC*	*GCTCACTTCACTCTCCCCAA*
Pdgfrb	*ACAACAACTCACTAGGGCCG*	*ATGTAGCGTCACCTCCAGC*
Ebf1	*cAGGAAACCCACGTGACATG*	*TGTTCCAGATAAGAGGGCGT*
Cthrc1	*CTGCTCGGTCTCTTCCTTGT*	*CCATCACGACCGGGAACT*
Ccl2	*CCACTCACCTGCTGCTACT*	*CTTCTTGGGGTCAGCACAGA*
Ccl11	*CCCAACTTCCTGCTGCTTTAT*	*ACTTCTTCTTGGGGTCAGCA*
Ccl7	*CATGCTGCTATGTCAAGAAACA*	*TCCTCGACCCACTTCTGATG*
Cxcl12	*CCTGAGCTACCGATGCCC*	*TTCGGGTCAATGCACACTTG*

Table S2. Antibodies used for Western blot (WB), Immunohistochemistry (IHC) and Immunofluorescence (IF).

Antibody	Reference	Applications	Dilution
PRRX1	Sigma (SAB1412737)	WB	1/1500
PRRX1	Sigma (ZRB2165)	IHC	1/800
PI16	Bio-Techne (AF4929)	IHC	1/1500
CTHRC1	Sigma (MABT889)	IHC	1/800
COL1	Southern Biotech (1310-01)	WB	1/2500
FN1	Abcam (ab2413)	WB	1/2000
ACTA2	Sigma (A5228)	WB / IF / IHC	1/10000
TUB	Abcam (ab2413)	WB	1/10000
RFP	Rockland (039600-401-379)	WB / IHC / IF	WB (1/5000) / IHC and IF (1/2000)
HSC70	Santa Cruz (SC-7298)	WB	1/2000
Vimentin	Abcam (ab92547)	IF	1/2000
PDGFRα	Cell signaling (3174S)	IF	1/800
CD45	Sigma (ZRB1180)	IF	1/800
CD31	Abcam (ab134168)	IF	1/400
NG2	Abcam (ab 183929)	IF	1/400
CC10	Merck (07-623)	IF	1/800

Disease Models & Mechanisms • Supplementary information
